# Integrative analysis of the methylome and transcriptome of tomato fruit (*Solanum lycopersicum* L.) induced by postharvest handling

**DOI:** 10.1093/hr/uhae095

**Published:** 2024-03-25

**Authors:** Jiaqi Zhou, Sitian Zhou, Bixuan Chen, Kamonwan Sangsoy, Kietsuda Luengwilai, Karin Albornoz, Diane M Beckles

**Affiliations:** Department of Plant Sciences, University of California, Davis, One Shields Avenue, CA, USA; Department of Plant Sciences, University of California, Davis, One Shields Avenue, CA, USA; Department of Biostatistics, School of Public Health, Columbia University, 722 West 168th Street, New York, NY 10032, USA; Department of Plant Sciences, University of California, Davis, One Shields Avenue, CA, USA; Germains Seed Technology, 8333 Swanston Lane, Gilroy, CA 95020, USA; Department of Horticulture, Faculty of Agriculture at Kamphaeng Saen, Kasetsart University, Kamphaeng Saen Campus, Nakhon Pathom 73140, Thailand; Department of Horticulture, Faculty of Agriculture at Kamphaeng Saen, Kasetsart University, Kamphaeng Saen Campus, Nakhon Pathom 73140, Thailand; Department of Plant Sciences, University of California, Davis, One Shields Avenue, CA, USA; Department of Food, Nutrition, and Packaging Sciences, Coastal Research and Education Center, Clemson University, 2700 Savannah Highway, Charleston, SC 29414 USA; Department of Plant Sciences, University of California, Davis, One Shields Avenue, CA, USA

## Abstract

Tomato fruit ripening is triggered by the demethylation of key genes, which alters their transcriptional levels thereby initiating and propagating a cascade of physiological events. What is unknown is how these processes are altered when fruit are ripened using postharvest practices to extend shelf-life, as these practices often reduce fruit quality. To address this, postharvest handling-induced changes in the fruit DNA methylome and transcriptome, and how they correlate with ripening speed, and ripening indicators such as ethylene, abscisic acid, and carotenoids, were assessed. This study comprehensively connected changes in physiological events with dynamic molecular changes. Ripening fruit that reached ‘Turning’ (T) after dark storage at 20°C, 12.5°C, or 5°C chilling (followed by 20°C rewarming) were compared to fresh-harvest fruit ‘FHT’. Fruit stored at 12.5°C had the biggest epigenetic marks and alterations in gene expression, exceeding changes induced by postharvest chilling. Fruit physiological and chronological age were uncoupled at 12.5°C, as the time-to-ripening was the longest. Fruit ripening to Turning at 12.5°C was not climacteric; there was no respiratory or ethylene burst, rather, fruit were high in abscisic acid. Clear differentiation between postharvest-ripened and ‘FHT’ was evident in the methylome and transcriptome. Higher expression of photosynthetic genes and chlorophyll levels in ‘FHT’ fruit pointed to light as influencing the molecular changes in fruit ripening. Finally, correlative analyses of the -omics data putatively identified genes regulated by DNA methylation. Collectively, these data improve our interpretation of how tomato fruit ripening patterns are altered by postharvest practices, and long-term are expected to help improve fruit quality.

## Introduction

Postharvest handling approaches are commonly used to extend tomato fruit shelf-life. Examples of these approaches include [1] harvesting fruit before full maturity, [2] refrigeration, [3] chemical treatments like calcium chloride or 1-MCP to inhibit ethylene production and [4] applying modified atmospheres with varying oxygen (O_2_) and carbon dioxide (CO_2_) proportion [1, 2] to suppress or inhibit ripening physiology. Ethylene can be applied at the end of postharvest storage to accelerate ripening or achieve uniform ripening for better marketing [3]. However, while a longer shelf-life benefits the produce trade by reducing fruit deterioration and postharvest loss, the unintended negative effects on fruit quality can lead to rejection by the consumers, creating postharvest waste [4]. Understanding the mechanisms of postharvest-induced changes in tomato fruit physiology and molecular biology is a first step toward finding a solution for postharvest loss and waste [5].

Harvesting tomato fruit before full ripening is an efficient approach to extend their shelf-life. However, the lack of energyand nutrient support from the mother plant often causes off-the-vine fruit to be suboptimal in quality, negatively influencing fruit sugar-to-acid ratio, volatile profiles, texture, and weight [6–9]. Depending on the postharvest storage conditions, i.e. temperature, light, dark, humidity, carbon dioxide, and oxygen concentration, fruit ripening and the development of quality traits are differentially affected [2]. Conversely, fruit ripened on the vine can import sugars and other compounds for an extended time and be exposed to a longer period of sunlight, which is important to fruit quality [10].

Low temperature storage is also used to slow down senescence and preserve quality in harvested fruit by reducing the rate of respiration biochemical reactions, fungal infestation, and water loss [5]. Conversely, tomato and other tropical and subtropical crops are sensitive to cold. Postharvest chilling injury (PCI) widely occurs when sensitive produce are stored at temperatures below the threshold [3, 11, 12]. Tomato fruit stored below 12.5°C may show symptoms of PCI upon rewarming to room temperature, such as abnormal firmness and texture, uneven ripening, fruit surface pitting, and spoilage from fungi [13]. The severity of PCI symptoms depends on the time–temperature combination and preharvest factors [14].

The current understanding of the molecular basis of fruit development, ripening, and senescence is highly developed in tomato, even if there remain many unanswered questions. The regulation of fruit ripening mechanisms not only focuses on hormones, mainly ethylene but also in abscisic acid (ABA), jasmonic acid, cytokinin, gibberellins, and auxin in recent years [15–18]. The rapid increase in ethylene is a well-established and critical feature of climacteric fruit ripening [19–21], but recently, evidence for ABA has been discovered [22, 23]. The mechanism of hormone interplay, including that between ABA and ethylene in fruit ripening, is still unclear. The current hypotheses are that (i) ABA may collaborate with ethylene signaling to activate tomato fruit ripening [24] and (ii) ABA might act upstream of ethylene signaling because ABA peaks before ethylene climacteric burst [25], and exogenous ABA could activate ethylene biosynthesis genes like *ACSs* and *ACOs* [26]. Further, although ABA is ‘the stress hormone’, ethylene, like ABA, is responsive to unfavorable changes in environments. However, the crosstalk among the ABA- and ethylene-mediated signal transduction pathways and their influence under postharvest chilling remain unclear.

A critical role for DNA demethylation in governing tomato fruit ripening and hence quality has also been recognized. Demethylation events occur at the promoter regions of ripening genes, presumably controlling transcription factor (TF) binding, thereby dictating if genes will be turned on/off [27]. Active DNA demethylation is enacted by DNA glycosylases, of which SlDML2 is the most important in tomato, as silencing *SlDML2* halts ripening [28]. Chilling stress inhibits *SlDML2* expression, suppressing ripening-associated demethylation; however, this action is partially reversed when fruit are rewarmed [29]. Changes in tomato fruit DNA methylation levels due to chilling correlate with flavor loss and variation in the transcriptional levels of key ripening genes [30]. Other epigenetic modifications also affect DNA demethylation [31], and this epigenome remodeling can collectively change fruit shelf-life and quality [8, 32].

The widespread reprogramming that occurs during ripening can be explored using -omics scale research, where multiple biological pathways can be simultaneously explored to systematically unravel the underlying mechanisms [33]. Transcriptomic analysis has enabled an understanding of key ripening pathways under varied postharvest conditions [32]. DNA methylomics analysis can precisely pinpoint changes in methylation status at loci under certain conditions. Individually, −omics studies like transcriptomics and methylomics can be used to explore global differences and generate co-expression networks with key markers highlighted across treatments [34]. Integrating these data can lead to the discovery of correlations among epigenetic and transcriptional changes, pointing out potential regulatory mechanisms of key biological processes [35].

In this work, we studied how postharvest handling, i.e. off-the-vine ripening and low-temperature storage affect tomato ripening and quality, by accessing the fruit transcriptome and methylome and studying ripening hormones and physiological traits. Comparisons were made on fruit at the same developmental stage but that underwent different postharvest storage simulating conditions used in industry. Integrative analysis was used to connect fruit ripening physiology and events at the epigenomic and transcriptomic levels. Our work may identify potential postharvest biomarkers, i.e. differentially expressed, or methylated genes that correlate strongly with, and are indicative of a particular postharvest treatment or fruit quality state, which may be useful for diagnosis and commercialization. Postharvest biomarkers would also be good targets for genome or epigenome editing for future fruit improvement.

## Results

### Postharvest treatments induced variations in fruit quality and methylome

**Figure 1:**
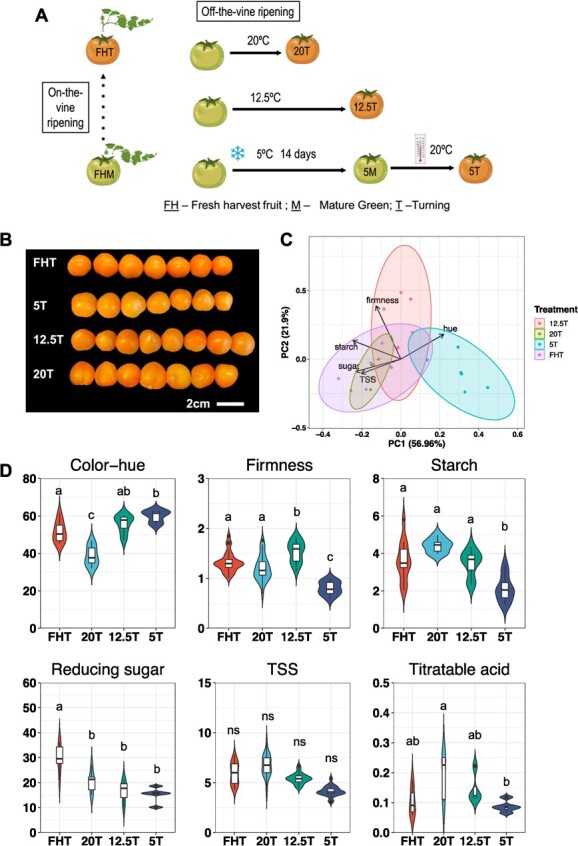
Postharvest treatments and fruit quality. (**A**) Postharvest fruit experimental design (adapted from Zhou *et al*. [8]). The time taken for fruit harvested at ‘MG’ fruit to reach Turning (‘T’) is indicated as the relative length of the black solid lines. (**B**) Photos of tomato fruit at the Turning stage after different postharvest treatments. (**C**) Principal component analysis (PCA) of the fruit quality parameters, with loadings. (**D**) Individual fruit quality parameters including hue angle (°), firmness (g), starch (mg. starch g^−1^ FW), reducing sugar (mg. sugar g^−1^ FW), total soluble solids (TSS) (°Bx), and titratable acid (TA) (meq. 100 g^−1^ FW). Tukey’s multigroup tests were applied and the letters above each bar indicate the significance levels, while ‘ns’ indicates no difference (*P* > 0.05).

Fruit were harvested at mature green (MG) and allowed to ripen at 20°C, 12.5°C, 5°C, and 5°C plus rewarming at 20°C, as described previously [8]. There were two MG groups, i.e., fruit fresh-harvested at MG (‘FHM’), and ‘FHM’ stored at 5°C for 2 weeks (‘5M’). There were four Turning fruit groups: three were ripened postharvest, i.e. fruit were harvested at ‘FHM’ and then stored at 20°C (‘20T’), 12.5°C (‘12.5T’), and 5°C plus rewarming at 20°C (‘5T’), and the fourth group consisted of fruit that were freshly harvested after they reached Turning (‘FHT’) on-the-vine (Fig. 1A).

Quality traits assessed in the fruit samples at the Turning stage included objective color, reducing sugars, total soluble solids, starch, titratable acids, and firmness [8]. Although the fruit from different postharvest treatments looked similar (Fig. 1B), this similarity in apparent color hid variation in quality, as shown in Fig. 1C and D. ‘FHT’ and ‘20T’ fruit were highly similar (they overlapped on the PCA plot). The ‘12.5T’ fruit were intermediate to ‘5T’ and ‘FHT’ on the plot, mainly due to its high firmness (*P* < 0.05). The ‘5T’ was distinct to ‘FHT’, and presumably had the worst quality profile from others, as it had lower contents in all traits, except color.

**Figure 2:**
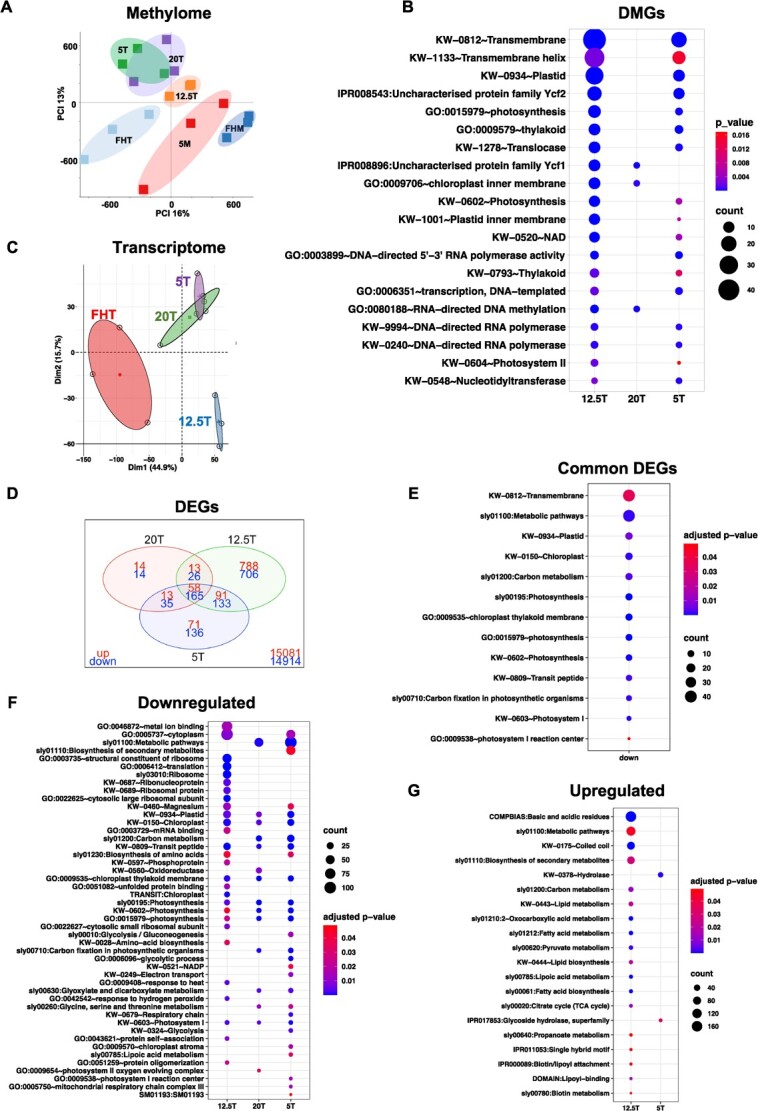
Analysis of the postharvest tomato fruit methylome and transcriptome. (A) Principal component analysis (PCA) of the fruit methylome. (B) Annotation of DMGs in pairwise comparisons, using ‘FHT’ as the control. The comparisons are (i) ‘12.5T’ vs ‘FHT’, (2) ‘20T’ vs ‘FHT’, (3) ‘5T’ vs ‘FHT’. The adjusted *P* <0.05 was used as the threshold and gene numbers in each term are indicated by ‘count’. For the ‘12.5T’, the terms that overlapped with either ‘5T’ or ‘20T’ are presented in this plot, and other unique terms are in the Fig. S15. (C) PCA of the transcriptome in ‘Turning’ fruit, i.e. ‘FHT’, ‘20T’, ‘12.5T’ and ‘5T’. (D) Venn plot of the DEGs in pairwise comparisons. The numbers in top (red )and bottom (blue) represent upregulated and downregulated DEGs compared to ‘FHT’, respectively. (E) Enrichment analysis of common DEGs (postharvest fruit compared to ‘FHT’) using DAVID (adjusted *P* <0.05) was shown, and they are all from downregulated genes. (F–G) when the postharvest Turning, i.e. ‘12.5T’, ‘20T’ or ‘5T’ was compared to the ‘FHT’, the representative terms from DAVID (adjusted *P* < 0.05) for downregulated genes were shown in (F) and upregulated genes in (G).

To determine the influence of various postharvest treatments on fruit methylation, context-specific methylation levels were assessed (Figs. S1–S3, Tables S1). The methylome of green fruit (‘FHM’ and ‘5M’) was similar to each other and distinct from Turning fruit (Fig. 2A). Within the Turning fruit, those ripened postharvest, i.e. ‘20T’, ‘12.5T’, and ‘5T’, clustered away from ‘FHT’, suggesting that ripening after harvest, regardless of storage temperature, affects the fruit methylome.

When comparing the quality and DNA methylation PCA (Figs 1C and 2A), incongruity was seen between ‘FHT’ and ‘5T’. ‘FHT’ has similar quality traits as the off-the-vine ripening ‘20T’ but a different methylation profile, whereas ‘5T’ had a similar methylation status to ‘20T’ but distinctly lower quality. We anticipated greater methylation marks on genes in cold-stored fruit, and the ‘12.5T’ would have similar methylome to other Turning fruit, but in contrast, our data showed that ‘12.5T’ was very similar to ‘5M’ (Figs. 2A and S3A).

### Differentially methylated genes and differentially expressed genes consistently associated with photosynthetic activities

To understand the DNA methylation differentiation due to treatment, pairwise comparisons were performed, and the differentially methylated regions (DMRs) were identified (Table S2). By comparing each postharvest ripened fruit to the ‘FHT’, i.e. (1) ‘5T’ (2) ‘12.5T’ and (3) ‘20T’, DMRs due to off-the-vine ripening at the respective temperatures could be inferred (Fig. S3). Further, the differentially methylated genes (DMGs) among postharvest Turning fruit compared to ‘FHT’ were extracted (Table S3). The DMRs analysis showed that the ‘12.5T’ fruit were the most unusual, with the highest number of DMGs and DMRs (most hypermethylated), compared to ‘FHT’ (Fig. S3).

The DMGs analysis using DAVID [36] indicated that ‘transmembrane’, ‘plastid’, ‘photosynthesis’, and ‘RNA polymerase’ were significantly enriched, when ‘12.5T’ and ‘5T’ were compared to ‘FHT’, respectively (Fig. 2B). The terms ‘plastid’ and ‘photosynthesis’ imply that low temperature regulates genes during the fruit chloroplast to chromoplast transition may be modulated by DNA methylation. The ‘20T’ has the least DMGs compared to others, leading to a limited number of enriched terms, with ‘chloroplast’ notably present (details in Tables S3 and S4 and Fig. S15).

Variation in gene methylation may have consequences for gene expression and downstream physiological processes. To examine this, we profiled changes in the tomato fruit transcriptome. RNASeq analysis indicated that 16 129 genes were expressed in fruit. We focused on the fruit ripened postharvest and compared them to fruit ripened on the vine (‘FHT’). Postharvest ripened fruit were more like each other and differed from ‘FHT’ (Figs 2A and S4). Although the fruit ripened after cold storage, i.e. ‘5T’, had quality traits that differed from ‘20T’ (Fig. 1), when comparing their mRNAs, these fruit were very similar, because the effects of the prior chilling event on the transcriptome were erased after rewarming [8, 29].

The differentially expressed genes (DEGs) in pairwise comparisons were identified using a criterion of 2-fold expression changes and adjusted *P-*value <0.01 (Table S5). The ‘12.5T’ had the largest number of DEGs (1030 up and 950 down) compared to all other groups (Fig. 2D). The ‘20T’ fruit were similar to ‘FHT’, having the lowest number of DEGs, most likely related to early harvest and dark storage treatments. The trend of DEG numbers was consistent with the DNA methylation data for these fruit.

Enrichment analysis of the common DEGs (58 up- and 165 downregulated in Fig. 2D) for all postharvest Turning groups compared to ‘FHT’ was shown in Fig. 2E (details in Tables S6 and S7, Fig. S16). Of note is that there was no significant term emerging from the 58 upregulated genes. Many photosynthesis-associated pathways were downregulated in the postharvest-ripened compared to the ‘FHT’ fruit (Fig. 2E, F). In addition, the genes associated with ‘carbon metabolism’ were enriched (Fig. 2E), specifically, *beta-amylase 8,* which was differentially expressed among Turning fruit. High *beta-amylase 8* expression in all postharvest fruit compared to ‘FHT’ was also validated by RT-qPCR (Fig. S23), indicating that starch degradation may be more active during the off-the-vine ripening process, which corresponds to the reduced starch seen in the postharvest fruit [8] (Fig. 1D).

The shared or unique down- or upregulated gene-terms across fruit groups were examined (Fig. 2F, G). ‘12.5T’ fruit, with the highest number of DEGs, had the most unique terms. The downregulated DEGs of ‘12.5T’ were enriched for ‘translation’, ‘ribosomal’, and ‘phosphoprotein’, indicating the importance of the post-translational modifications in ‘12.5T’ relative to ‘FHT’. The upregulated DEGs of ‘12.5T’ were enriched in terms for metabolic processes and primary and secondary metabolites. There were no upregulated terms found in ‘20T’, indicating similarities with fruit ripened on the vine (‘FHT’).

The analysis of DEGs and DMGs collectively indicate that (i) physiological alterations in energy capture and use occurred in postharvest-ripened compared to vine-ripened fruit; (ii) potential correlations between DNA methylation and gene expression exist, with possible ensuing effects on fruit metabolism (Tables S10 and S11); (iii) the low but non-chilling temperature storage (‘12.5T’) led to great changes in the methylome and transcriptome, although the fruit had the same objective color and ripening characteristics as other Turning fruit.

### Gene co-expression network by WGCNA

We used weighted gene co-expression analysis (WGCNA) to identify gene modules related to specific postharvest storage conditions. The DEGs from the comparisons of postharvest Turning (i.e. ‘20T’, ‘12.5T’, ‘5T’) to the fresh-harvested Turning (‘FHT’) were pooled together. The 2255 unique genes as the input dataset were clustered as six module eigengenes (ME), i.e. turquoise (993 genes), blue (539), brown (358), yellow (182), grey (128), and green (55) (Figs S5–S12).

The ME turquoise and ME blue modules were distinct (Fig. S4). ME turquoise genes were strongly and positively correlated in ‘FHT’ (*r* = 0.82, *P* < 0.001), but no correlation was seen in the postharvest-ripened fruit. Genes in ME blue were positively correlated in ‘12.5T’ (*r* = 0.78, *P* < 0.001) but negatively correlated in ‘FHT’ (*r* = −0.62, *P* = 0.01). The genes in ME brown were negatively correlated in all postharvest fruit but positively related in the ‘FHT’ fruit (Fig. S5). Overall, these data reinforce the divergence in gene expression between ‘FHT’ and postharvest fruit (Fig. 2C), especially with ‘12.5T’.

The genes in each ME were annotated using GO terms [37] and DAVID (Figs S13 and S14, Table S9). With DAVID, (i) only genes in ME blue, brown, and turquoise had significant terms; (ii) the genes in ME brown were associated with ‘plastid’, ‘chloroplast’, and ‘photosynthesis’; (iii) the ME turquoise module had top terms such as ‘amino-acid biosynthesis’ and ‘response to heat’, and (iv) in ME blue, terms such as ‘cytoplasm’, ‘carbon metabolism’, and ‘fatty acid’ were prominent.

Analysis of the gene network of each module (Figs S7–S12) can help to identify ‘hub genes’, i.e. those highly connected to others (Table S8). These hub genes potentially work upstream in the fruit transcriptomic response to postharvest treatments, making them good candidates to study postharvest fruit ripening biology [38].

### Fruit carotenoids and ABA content

**Figure 3:**
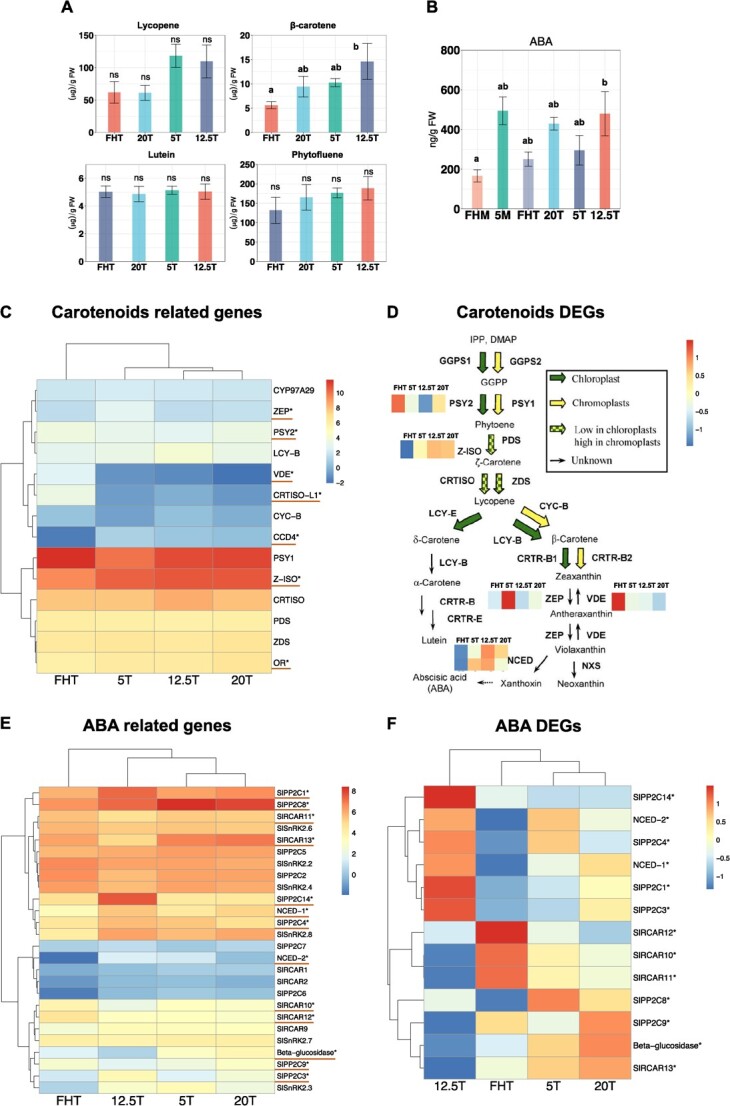
Metabolite and transcriptomic analysis of fruit carotenoids and abscisic acid (ABA). Metabolite levels of fruit (A) carotenoids - lycopene, lutein, β-carotene and phytofluene and (B) ABA contents. The error bars represent the standard deviation of the mean of three biological replicates, except for ‘5M’ which only has two replicates in the ABA assay. The Tukey’s multigroup tests were applied. The letters above each bar indicate the significance levels, and ‘ns’ indicate no difference (*P* > 0.05). (C) Transcriptomic analysis of the carotenoids related genes. This heatmap was generated by the Log_2_ (Counts per million-CPM). Tukey’s multigroup tests were applied and asterisks and red lines were added only for the DEGs (*P* < 0.05), without filtering by gene expression fold-change. This method was applied to all gene expression heatmaps below. (D) Transcriptomic analysis of the carotenoids biosynthetic pathway adapted from Galpaz *et al*. [39]. The DEG expression heatmaps were annotated on the side of the pathway. We use the zoomed color scale, from −1 to 1, to highlight subtle changes in gene expression for the DEGs. (E) Transcriptomic analysis of all expressed ABA-related genes (F) Heatmaps of ABA related DEGs in (E) using the zoomed color scale.

We next aimed to connect changes in molecular events, i.e. mRNA and DNA methylation with biochemical and physiological processes related to ripening. Fruit carotenoids, including lycopene, β-carotene, lutein, and phytofluene were assessed in Turning fruit. The ‘12.5T’ fruit had relatively high carotenoids, and uniquely, its β-carotene content was 2.6-fold higher than ‘FHT’ (Fig. 3A). There was high within-group variability in the carotenoids data, indicating strong interactions of pre- and postharvest factors on metabolite content [40].

Transcriptome analysis of the carotenoid-related pathway showed that *Z-ISO*, which is upstream of β-carotene synthesis, was upregulated in ‘12.5T’ fruit (Fig. 3C and D). This may explain the high contents of β-carotene in ‘12.5T’. The enzymes encoded by *ZEP* and *VDE* inversely regulate β-carotene metabolism [41]. *ZEP* was upregulated in ‘5T’—2.3-fold versus ‘FHT’, and *VDE* was upregulated in ‘FHT’—15.0-fold versus ‘12.5T’. However, post-transcriptional regulation of carotenogenic enzymes may lead to nonlinear connections between gene expression and carotenoid content.

ABA is produced downstream of the carotenoid biosynthesis pathway as a stress-responsive and ripening-related hormone (Fig. 3D). Fruit ABA increases from immature green to Turning, then decreases until red ripe [26], we therefore included green fruit in this analysis. In accordance, all Turning fruit had higher ABA content than ‘FHM’ (Fig. 3B). With ‘FHM’ as the control, the ‘12.5T’ fruit had more ABA (2.9-fold) accumulated than other Turning fruit (i.e. 1.5-fold in ‘FHT’, 2.6-fold in ‘20T’, 1.8-fold in ‘5T’). We examined the RNASeq data for connections between ABA content and transcription. The rank of ABA content was ‘12.5T’ > ‘20T’ > ‘5T’ > ‘FHT’, and, expression of *NCED-1*, the rate controlling gene for ABA biosynthesis, showed the same trend as ABA content (Fig. 3E and F). The uniformly high ABA contents and ABA biosynthesis gene expression in stored fruit may indicate an ABA-stress response activated by early harvest and postharvest storage.

We extracted the DEGs (Fig. 3F) from all expressed ABA genes in Fig. 3E, and the ‘12.5T’ expression pattern was unique among all Turning fruit. In ‘12.5T’, both *NCED* isoforms were expressed highest compared to ‘FHT’; *NCED-*1 was 3.9-fold and *NCED-*2 was 10.2-fold higher. However, the *beta-glucosidase* gene that can release free ABA by hydrolyzing ABA-GE [42], was downregulated in ‘12.5T’. It is plausible that this gene is inhibited due to saturated ABA levels in ‘12.5T’ fruit. All four ABA receptor genes were suppressed, i.e. *SlRCAR13* (also named *SlPYL1* [43]*), SlRCAR12, SlRCAR10*, and *SlRCAR11.* Expression of some *protein phosphatases 2C* (*PP2C*) involved in ABA signaling was remarkably high in ‘12.5T’ fruit. These data indicate that in addition to early harvest, low temperature stress over a prolonged period may induce a sustained ABA stress response, which was tracked with higher levels of ABA and the complicated transcriptional regulation of the genes in ‘12.5T’.

### Postharvest fruit ethylene production and respiration rates

**Figure 4:**
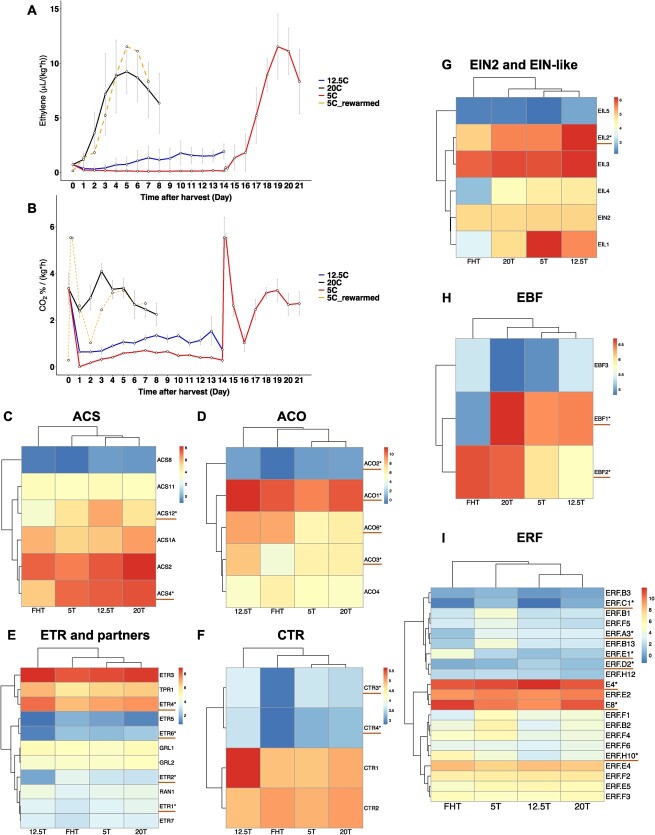
Ethylene and carbon dioxide production in relation to gene expression in the postharvest fruit. (A) Ethylene production and the (B) CO_2_ levels of the fruit harvested at the MG and stored at 20°C (black line), 12.5°C (blue line), and 5°C (red line) for 2 weeks and rewarmed to 20°C (red line). The rewarming trendline was moved to the same x-axis scale (shown as the dashed orange line) to compare with ‘20C’. The error bar represents standard deviation of the mean of the six biological replicates used in this assay. Tukey’s multigroup statistical tests were performed as shown in Table S14. (C–I) Ethylene biosynthesis and related gene expression heatmaps by gene families: (C) *ACS* (1-aminocyclopropane-1-carboxylic (ACC) synthase, (D) *ACO* (ACC oxidase), (E) *ETR* (ethylene receptors) and partners, (F) *CTR* (constitutive triple response), (G) *EIN* (ethylene-insensitive)-2 and EIN-like, (H) *EBF* (EIN3-binding F-box), (I) *ERF* (ethylene response factor). Both the asterisks and red line were added only for the DEGs (*P* < 0.05). The gene lists and ID are according to previous study [45].

Ethylene and carbon dioxide (CO_2_) production are characteristic of climacteric fruit ripening, and changes in the rate of production also serve as stress biomarkers for postharvest tomato ripening [14, 44]. Ethylene production and respiration rates from MG until fruit ripening were depicted in Fig. 4A and B. The ethylene produced by ‘5M’ after rewarming was projected (dashed lines) onto the same timescale of the 20°C stored fruit, allowing comparisons between normal fruit ripening and stress-response-related ripening. First, total ethylene production under 20°C and 5°C rewarmed were similar (Table S14), indicating that chilling did not change the amount of ethylene produced but induced differences in production rates. Second, the rewarmed fruit had the characteristic intense burst of ethylene compared to normal ripening (20°C) (Figs 4A and S20), indicating stress induced rapid ethylene accumulation. This sharp ethylene burst could trigger physiological decay of fruit quality compared to the normal ripening.

There were two peaks of respiratory activity in the rewarmed fruit (Fig. 4B). The first peak at Day 14 was likely the immediate stress response to increase metabolic activity for chilling injury recovery [46]. The second peak at the Days 18–19 occurred along with the ethylene burst, which is the typical climacteric fruit respiratory burst [47]. After Day 4, total CO_2_ production in the rewarmed fruit was close to that produced during normal ripening, indicated by the overlapping black and orange lines (Fig. 4B). In addition, Day 0 for all postharvest fruit showed the highest respiratory rates, which could be due to the stress after harvest.

Strikingly, the 12.5°C fruit showed no obvious climacteric ripening peak of ethylene or CO_2_ over the 14-day storage, even though the fruit at this temperature underwent normal color development and quality changes [8]. Furthermore, the 12.5°C fruit had reduced ethylene and CO_2_ total production compared to ‘20°C’ and ‘5°C_rewarmed’ during storage periods, even though the fruit were stored for 14 days (Table S14).

The noteworthy question is whether ethylene is the hormone driving apparent fruit ripening under 12.5°C. We, therefore, looked at the expression of genes involved in the ethylene pathways (Fig. 4C–I). In tomato, there are two systems responsible for ethylene production, system 1 is autoinhibited producing limited amounts of ethylene, while system 2 is autocatalytic and responsible for fruit ripening [45]. There were no differences in gene expression for system 1 ethylene [45] in our postharvest fruit, i.e. *ACS1A* was universally expressed (Fig. 4C) and *ACS6* was not expressed. The transition to system 2 ethylene depends on *ACO1* and *ACO4*; *ACO1* expression in ‘12.5T’ was the highest compared to all other groups (Fig. 4D). This is possibly due to ABA induction, considering the high ABA content in ‘12.5T’ fruit [26]. The genes mediating system 2 ethylene production include *ACS2*, *ACS4*, *ACS1A*, *ACO1*, and *ACO4*, of which, *ACS4* was upregulated in all postharvest groups, while *ACO1* was downregulated in ‘5T’.

Our ethylene signaling pathway data suggest the following: (i) the main ethylene receptor genes, *ETR4* and *ETR3* (also named *NR*), were highly expressed in the ‘12.5T’ fruit (Fig. 4E). *ETR4* repression resulted in faster fruit ripening [48, 49]. (ii) Ripening-related *CTRs* (3 and 4), negative regulators of ethylene signaling transduction, were downregulated in ‘FHT’ only (Fig. 4F). (iii) The DEGs of other ethylene-related gene families, such as *EIN*, *EBF*, and *ERF* (Fig. 4G–I), were highlighted, although some were expressed at low levels or are less studied. (iv) The ethylene responsive factors *E4* and *E8* are ethylene and ripening-induced [50] and were extensively expressed across all groups (Fig. 4I). Specifically, *E4* showed the highest expression in ‘12.5T’ fruit, while ‘FHT’ had the highest *E8* expression. (v) A known ethylene responsive factor *ERF.E1* [51] was only upregulated in ‘FHT’ (Fig. 4I).

In summary, the ethylene transcriptomic analysis illustrated the observed discrepancy and complexity between ‘12.5T’ and ‘FHT’ fruit, suggesting that 12.5°C storage delays the typical expression changes during fruit ripening. The ‘12.5T’ fruit had relatively low ethylene levels, no obvious ethylene system 2 peak but unique expression profiles of some ethylene-related genes (*ACS12*, *ETR2*, *ETR4*, *ETR6*, *EIL2*, *EBF2* etc.). The mechanisms underlying these surprising findings may be related to the enhanced ABA in ‘12.5T’ fruit (a proposed model is presented in Fig. 6B).

### Fruit photosynthetic-related activity

**Figure 5:**
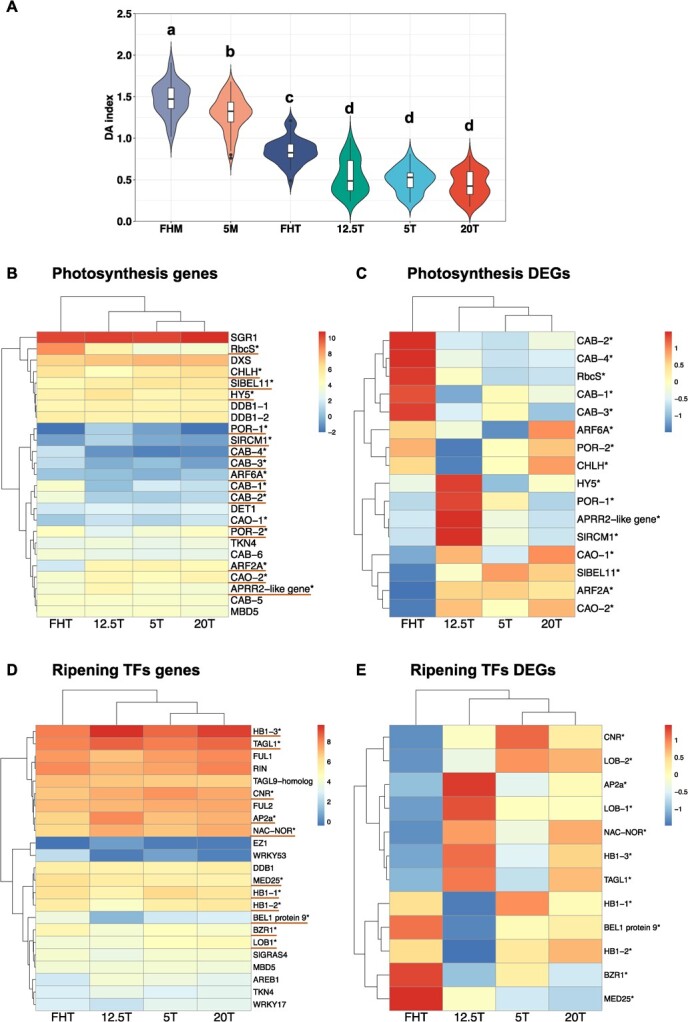
Postharvest tomato fruit *I*_DA_ in relation to photosynthetic genes expression. **(A)** Postharvest fruit *I*_DA_. Each treatment includes 20 individual fruit as biological replicates. Tukey’s multigroup tests were applied and the letters above each bar indicate the significance levels (*P* < 0.05). (**B**) Expressed fruit photosynthetic-related genes heatmap using Log_2_ CPM. (C) DEGs were extracted from (B), with expression zoomed from −1 to 1. (D) Fruit ripening transcription factors (TFs) expression using Log_2_ CPM. (E) DEGs were extracted from (D), with a zoomed color scale from −1 to 1. Both the red lines and asterisks indicate the DEGs (*P* < 0.05).

The role of photosynthesis during tomato fruit ripening has been underestimated but was highlighted by the methylome and transcriptome data in this work. To determine if there was an association between the -omics data and the fruit photosynthetic markers, the delta absorbance (DA) index (I_DA_) was assessed. As expected, the MG fruit had a higher I_DA_ than the Turning fruit (Fig. 5A). Specifically, among the Turning fruit, the ‘FHT’ had the highest I_DA_ values compared to all others.

**Figure 6:**
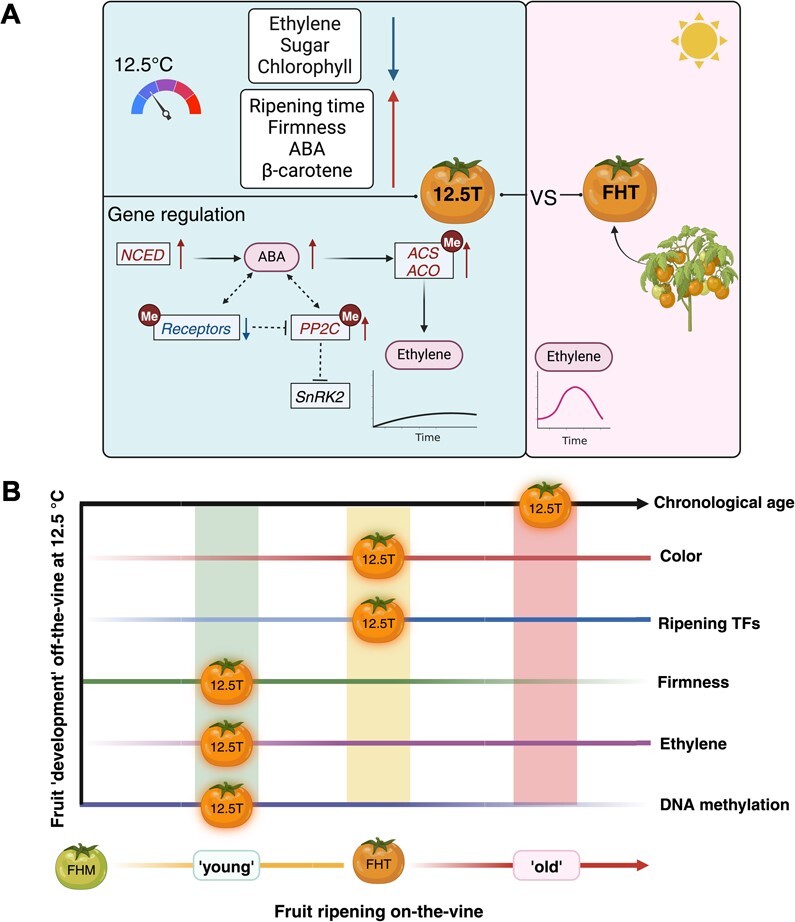
Proposed regulatory pattern for the ‘12.5T’ compared to the ‘FHT’ fruit. (A) Integrative perspective of fruit physiology and ripening hormones. Across the physiological traits assessed in this study, the ‘12.5T’ fruit exhibited: (a) reduced ethylene production, sugars, and I_DA_; (b) extended ripening time, high firmness, ABA levels, and β-carotene content (*P* < 0.05); and (c) color and other quality parameters (see Fig. 1) similar to the ‘FHT’. Hormone regulation: focusing on the ‘12.5T’ fruit, transcriptomic analysis suggests differential expression of ABA-related genes (Fig. 3F). We propose that upregulated *NCEDs* may lead to increased ABA production, and that storage at 12.5°C reduced expression of *RCARs*, potentially requiring more active ABA production to interact with receptor proteins. Contrary to typical ABA signaling transduction, our data showed activation of *PP2Cs*, and no changes in *SnRK2s*, implying an abnormal regulation of the ABA pathways. High ABA contents may contribute to the upregulation of ethylene biosynthesis genes [26], sustaining ethylene production under low temperatures postharvest. Furthermore, our data suggest that *RCARs, PP2Cs*, *ACOs*, and *ACSs* may be regulated by DNA methylation (see Table 2). (B) Chronological clocks versus multiple biological clocks in fruit off-the-vine development at 12.5°C. The chronological age of the ‘12.5T’ fruit does not align with its biological age. We use the term ‘development’ to describe the processes undergone by ‘12.5T’ fruit, recognizing it as more than a simple ripening and senescence process. We propose the existence of multiple biological clocks by integrating concepts elaborated by Jensen *et al*. in mammals [52] and by van de Poel *et al. *in tomatoes [53]. Using the ‘FHT’ fruit as the standard, our ‘12.5T’ fruit appears ‘young’ in the clocks of ‘firmness’, ‘ethylene’ and ‘DNA methylation’. However, it shared the same age under the clock of ‘fruit color’ and some master ‘ripening TFs’, i.e. *RIN*, *FUL1*, and *FUL2* expression and is evidently ‘older’ according to the chronological clock. This suggests a complex interplay of biological processes governing fruit development, under low but non-chilling temperature, with different traits exhibiting varied rates of changes over time.

 Transcriptomic analysis indicated that many photosynthesis-related genes were expressed at low levels in Turning fruit (Fig. 5B). It is worth noting that *SGR1*, a crucial gene in tomato chlorophyll degradation [54], was uniformly upregulated in all Turning fruit. *SGR1* is reported to be activated by fruit development and low temperature [55], suggesting that our postharvest treatments may not have a direct effect on chlorophyll degradation. When only focusing on DEGs (Fig. 5C), ‘FHT’ had remarkably high *CAB* genes expression. *CAB* are members of the chlorophyll a/b binding protein family, positively correlated with chlorophyll contents [56]. Chlorophyllide *a* oxygenase (CAO) catalyzes chlorophyll *a* to chlorophyll *b*, and this gene was downregulated in the ‘FHT’. *BEL11* and *ARF2A* are negative regulators of fruit chloroplast development and chlorophyll synthesis [57, 58], and they were upregulated in all postharvest fruit (Fig. 5C), which may be related to their reduced chlorophyll contents. The ‘FHT’ fruit had high expression of *CAB* and reduced *CAO*, *BEL11*, and *ARF2A*, which positively correlates to their high chlorophyll contents (Fig. 5A). Correlative analyses between (1) the I_DA_ and gene expression, and (2) DNA methylation and expression of photosynthetic genes were performed (Table 2). The expression of four genes was correlated (*P* < 0.05) with the I_DA_, two *CAB* genes, *CAO*, and *BEL11* (Fig. S22).

The dramatic changes in photosynthetic genes led to the next question, i.e. whether postharvest dark storage relates to the findings. To test this, we stored the MG fruit at 5°C under light or dark and the *I*_DA_ was assessed after 2 weeks. When compared to fresh harvested MG fruit, light-stored fruit at 5°C had the same *I*_DA_ as the ‘FHM’, while fruit stored under dark had lower *I*_DA_ values (Fig. S21).

### Correlative analysis on fruit ripening and quality pathways

We further examined the specific genes and regulatory factors involved in the ripening-to-senescence transition [59], e.g. genes involved in cell wall metabolism, auxin/IAA biosynthesis, fruit ripening TFs, and DNA methylation and histone regulation (Fig. 5D and E, S17–S19, Tables S12, S13 and S15) because of their importance to fruit postharvest quality. Their transcriptional levels and correlations between DNA methylation and gene expression were analyzed.

The expression pattern of some key ripening TFs showed similarity between postharvest fruit and ‘FHT’ (Fig. 5D). RIN, FUL1, and FUL2, which form a protein complex to regulate fruit ripening genes [60], were highly and similarly expressed in all groups. However, when DEGs are considered, Fig. 5E indicates that all postharvest ripened fruit had distinct profiles from ‘FHT’, but ‘12.5T’ fruit differed from ‘5T’ and ‘20T’. The five genes (*AP2a*, *LOB-1*, *NOR*, *HB1-3*, and *TAGL1*) in ‘12.5T’ were upregulated and three genes (*HB1-1*, *HB1-2*, and *BEL1 protein 9*) were suppressed compared to other groups. AP2a is a ripening and ethylene repressor [61], and the other genes, i.e. *LOB*, *NOR*, *HB1*, and *TAGL1* are positive ripening regulators [62]. *AP2a* expression in the ‘12.5T’ fruit indicated a complicated ripening transcriptional regulation.

Our correlative analysis points to genes with changes in DNA methylation at the promoter or within the gene body, which may be related to alterations in gene expression due to postharvest effects. There are ripening TFs, i.e. *HB1*, *MED25* [63], *NAC-NOR* and *WRKY17* [64], and *AP2a* [61] (Table 1), and many ethylene genes (Table 2). The two regions of the *NAC-NOR*, master ripening regulator in tomato, have inverse expression-methylation correlation, and its expression was remarkably high in ‘12.5T’ and ‘20T’. Histone deacetylases (HDAs), which control ripening by acting as transcriptional co-repressors [65]; their differential expression pattern in the ‘12.5T’ (Fig. S19C) may suggest regulation of histone deacetylation is affected by DNA methylation (Table 2).

**Table 1:** Ripening transcription factors with significant correlation between their DNA methylation and gene expression profiles

Gene name	PCC (*r*)	Correlation *p*-value	Methylation region
HB1–2*	0.6666	0.0179	Promoter
HB1–1*	−0.7750	0.0031	Gene body
MED25*	−0.7501	0.0050	Gene body
NAC-NOR*	−0.7192	0.0084	Gene body
*WRKY17*	−0.7106	0.0096	Gene body
HB1–1*	−0.6983	0.0116	Gene body
NAC-NOR*	0.6611	0.0193	Gene body
AP2a*	0.6361	0.0262	Gene body

There are four Turning groups (‘FHT’, ‘5T’, ‘12.5T’ and ‘20T’), and the DNA methylation data (with the CpG, CHG, and CHH contexts combined), and gene expression data were used for the analyses, each with three biological replicates. The *P*-value 0.05 was used as the threshold for both expression and correlative analyses. The same genes may have multiple Pearson’s Correlation Coefficients (PCC) due to multiple DNA methylation probes found in those gene associated regions. DEGs were indicated by a ‘*’ after gene name. Methylation region indicates where the probe locates to the gene.

**Table 2:** Genes involved in fruit ripening and fruit quality pathways with significant correlations found between their DNA methylation and gene expression status

Gene name	PCC (*r*)	Correlation *P*-value	Methylation region
Carotenoids-related			
VDE*	−0.7563	0.0044	Promoter
ZEP*	−0.8668	0.0003	Gene body
PSY2*	−0.7413	0.0058	Gene body
*PSY1*	0.6223	0.0307	Gene body
ABA-related			
PYL1*	−0.7191	0.0084	Promoter
Beta-glucosidase*	−0.8292	0.0009	Gene body
SlPP2C4*	0.7989	0.0018	Gene body
SlRCAR11*	−0.6926	0.0125	Gene body
SlRCAR10*	0.6089	0.0356	Gene body
Ethylene-related			
ERF.C1*	−0.7396	0.0060	Promoter
ACO3*	0.7085	0.0099	Promoter
ACO2*	−0.6881	0.0134	Promoter
*CTR1*	0.6056	0.0369	Promoter
ACO1*	0.8480	0.0005	Gene body
ERF.C1*	−0.8050	0.0016	Gene body
*CTR1*	0.7958	0.0020	Gene body
*TPR1*	0.7855	0.0025	Gene body
*ERF.B2*	−0.7148	0.0090	Gene body
ETR1*	−0.6809	0.0148	Gene body
EBF2*	−0.6807	0.0148	Gene body
ACS4*	−0.6250	0.0298	Gene body
*ERF.B3*	−0.6000	0.0392	Gene body
*ETR5*	−0.5844	0.0460	Gene body
*ERF.C.3*	−0.7423	0.0057	Gene body
Photosynthesis-related			
HY5*	0.8612	0.0003	Gene body
HY5*	0.7368	0.0063	Gene body
Auxin/IAA-related			
IAA22*	0.7731	0.0032	Promoter
SAUR51*	0.7408	0.0058	Promoter
IAA10*	0.7208	0.0082	Promoter
IAA8*	−0.6829	0.0144	Gene body
IAA13*	−0.6622	0.0190	Gene body
ARF7b*	0.5762	0.0499	Gene body
Cell wall-related			
PL*	0.8139	0.0013	Promoter
EXP1*	−0.7274	0.0073	Promoter
TBG3*	−0.8642	0.0003	Gene body
PL*	0.7616	0.0040	Gene body
TBG4*	−0.6369	0.0259	Gene body
DNA methylation and histone-related			
CMT3.1*	−0.8247	0.0010	Promoter
CMT2*	−0.7564	0.0044	Promoter
*RDR2*	0.7077	0.0100	Promoter
*JMJ6*	−0.5928	0.0422	Promoter
HDA1*	0.8520	0.0004	Gene body
*DML3*	0.7568	0.0044	Gene body
DRM2*	−0.7096	0.0097	Gene body
CMT3.1*	−0.7057	0.0103	Gene body
*DML1*	−0.6601	0.0195	Gene body
DRM2*	0.6491	0.0224	Gene body
AGO6*	−0.6201	0.0315	Gene body
*DML3*	0.6109	0.0348	Gene body
MET1*	0.5979	0.0400	Gene body
HDA5*	−0.6999	0.0113	Promoter
HDA3*	−0.8775	0.0002	Gene body
*HDA9*	0.6181	0.0322	Gene body

Analyses were done as described in the Table 1.

## Discussion

Our objective was to investigate the impact of early harvest combined with postharvest storage at different temperatures on fruit DNA methylation. We also aimed to assess whether these postharvest conditions led to significant changes in gene expression in fruit ripening pathways and fruit physiology. Our transcriptomic and methylomics data revealed striking differences between fruit ripened after harvest and those ripened on the vine, irrespective of temperature storage. Notably, photosynthesis genes were the primary determinants of this distinction. This is the first report that indicates substantial changes in the photosynthetic pathway in postharvest fruit. We also discovered that ‘12.5T’ fruit had the most distinctive DNA methylation and gene expression profiles, and it also displayed unique physiological traits, including carotenoids, ABA, and ethylene production.

Our work highlights significant changes in genes associated with ‘photosynthesis’ in postharvest fruit. The postharvest-stored fruit had reduced chlorophyll, supporting the clear distinction in the methylation status and expression of photosynthesis-associated genes. Fruit photosynthesis primarily depends on CO_2_ refixation from respiration, as well as active but limited chloroplast activity [66]. Many studies suggest that carbohydrates produced by fruit photosynthetic activity contribute to the energy and carbon required for synthesizing metabolites responsible for desirable fruit flavor attributes, maintaining O_2_ levels in the inner fruit tissue, and fueling seed development [67–69]. These discussions on the importance of fruit photosynthesis have focused on green fruit with active chloroplasts. During ripening, chloroplast degradation and the development of chromoplasts, accompanied by a decline in chlorophyll and an increase in carotenoids, limit fruit photosynthesis [70]. Our work is of note due to the upregulated photosynthetic transcriptional activity observed in Turning fruit on the vine compared to harvested fruit. This may underscore the significance of fruit photosynthetic activity during ripening. A recent study reported that fruit photosynthetic gene expression is upregulated in both green and ripened fruit under water stress when source capacity is constrained [71], indicating a dynamic tradeoff between source and sink photosynthesis to support organ development.

Our work points to the strong effect of light on the methylome, transcriptome, and chlorophyll levels of stored fruit compared to temperature and other stresses. Light is essential for fruit photosynthesis and chlorophyll synthesis [72, 73]. While chlorophyll captures light energy during photosynthesis, it may not always accurately predict photosynthetic activity. A proportional relationship between chlorophyll and photosynthetic rates may only occur under specific conditions and in certain plant tissue [74], although there is consistency in fruit chlorophyll contents, photochemical potential, and expression of photosynthesis related genes in Micro-Tom [75]. Therefore, whether light has a direct effect on postharvest fruit photosynthesis requires more evidence. It has been suggested that CO_2_ evolution rates are higher in dark-stored tomato fruit than in those stored in the light, possibly due to reduced photosynthesis [76]. Our data are suggestive and can be reinforced with measurements of net photosynthesis rates (change of CO_2_ levels), electron transport, and Rubisco activities, in addition to chlorophyll contents, to accurately indicate postharvest fruit photosynthetic activity.

Beyond the possibility of photosynthesis occurrence, evidence for light influencing fruit metabolism is numerous. Light (i) enhances respiration and induces an earlier onset climacteric ethylene peak, resulting in a shorter fruit shelf-life [77]; (ii) improves tomato nutritional quality and flavor [78]; (iii) controls fruit carotenoid development during ripening as an activation signal [79]; (iv) mediates signaling transduction associated with the methylation status of ripening genes’ promoters [80]. Taken together, these studies support that restricted light, a common practice in postharvest handling, may contribute to quality reduction in postharvest fruit.

The low but non-chilling storage of ‘12.5T’ fruit leads to distinctive profiles of DNA methylation and gene expression patterns, and carotenoid levels. Most interestingly, the ‘12.5T’ fruit had no ethylene climacteric burst but relatively high levels of ABA. Our hypotheses are that (i) this low temperature storage without rewarming suppressed the normal climacteric peak, and (ii) the complex hormone interplay of ethylene, ABA, IAA, GA, or others collectively lead to this biological ripening process [81]. Remarkably, since ABA is proposed to act upstream of ethylene in tomato ripening [24], an uncoupled ripening process may occur between ABA and ethylene in ‘12.5T’. Ethylene production in ‘12.5T’ may lag ABA production, leading to the unique molecular regulation observed in this work. Moreover, while there are reports on how chilling inhibits ripening and alters hormone interactions, few investigate the effects of low but non-chilling temperatures [82–84]. ABA receptors genes were suppressed in ‘12.5T’ fruit. Noticeably, *SlRCAR13* (*Solyc08g082180*) has a known role in postharvest fruit ripening. It is suppressed during postharvest cold storage in zucchini [85], and it is also downregulated in a long shelf-life tomato cultivar [86]. Therefore, the low expression of *RCARs* may be related to the slow ripening of fruit and high firmness. In addition, ‘12.5T’ showed inconsistent results in gene expression validation using RT-qPCR, but there was high similarity in results between the two methods, i.e. RNASeq and RT-qPCR, in all other groups (Fig. S23). These conflicting results indicate that pre-harvest environments across growth seasons significantly affect fruit gene expression after storage at 12.5°C [87]. This effect may be magnified because of the extended developmental program of these fruit, and near the chilling temperature threshold, chilling-related biological processes may be triggered sporadically.

We conducted a comparative study using two fruit stages, i.e. ‘Mature green’ and ‘Turning’. ‘Turning (T)’ is the ripening stage we selected for sampling and subsequent studies because (i) both the fruit stored at 5°C followed by rewarming and the fruit at 12.5°C consistently reached the ‘Turning’ stage but not red ripe, and (ii) in ‘Micro-Tom’, Turning corresponds to the ‘Pink’ that is the stage just before red ripe in conventional tomato cultivars [8, 88]. Studying the ‘Turning’ stage enables us to capture differential gene regulation associated with ripening and quality before fruit senescence which begins at red ripe. We compared postharvest fruit to the fresh harvest fruit with identical color attributes, which we used as a proxy for fruit developmental stage; however, there is a disconnect between the physiological and chronological age of fruit ripened postharvest. The ‘12.5T’ fruit that took the longest time to ripen from MG to Turning had the highest methylation levels among all the Turning fruit (Fig. S3). The fruit industry commonly uses color or other quality traits to define produce age. Instead, our data implied that the methylome indicated age may be more accurate than cellular or chronological age [89]. These fruit genomic molecular fingerprints could potentially serve as quality biomarkers for differentiating fruit internal quality parameters from external appearance, therefore, contributing to a reduction in postharvest waste in the future.

For our -omic studies, we used bulk sequencing, which indicates the average percentage of methylation and the average levels of gene expression across millions of cells. Correlative analysis between methylation and expression was established for known ripening genes, and the genes with significant correlation were highlighted (Tables 1, 2, S13 and S15). This information is important for crop improvement through epigenome engineering [90]. It is noteworthy that although we used low (3–4×) coverage of the tomato genome by bisulfite sequencing, the biological replicates remained consistent, and the methylation percentages closely aligned with results from a whole genome bisulfite sequencing (WGBS) study using single-base resolution [27]. Our study, along with the work of Crary-Dooley *et al*. [91] collectively supports the feasibility and reliability of low-coverage sequencing.

In conclusion, the analysis of -omics and physiological data in this work revealed that early harvest and storage have an impact on fruit ripening quality, hormone composition, and the transcriptome. Variations in many of these biological entities are closely associated with DNA methylation, as demonstrated by the expression-methylation correlations observed in many ripening genes. The integrative analysis of gene expression and DNA methylation correlation tests across multiple ripening and quality pathways pinpointed postharvest biomarker genes for future studies on tomato postharvest biology.

## Materials and methods

### Plant growth

*Solanum lycopersicum* L. cv. ‘Micro-Tom’, an experimental model cultivar for postharvest studies was used in this study. ‘Micro-Tom’ seeds were from the Tomato Genetics Research Center at UC Davis. Germination and plant growth methods were as described previously [8]. Postharvest treatments were done on fruit randomly harvested from over one hundred plants in 2020, 2021, and 2022.

### Fruit sampling and postharvest treatments

Fruit were sampled at two developmental stages: MG and Turning (T), as described by Zhou *et al*. [8] (Fig. 1). Harvested fruit were washed with 0.27% (v/v) sodium hypochlorite for 3 min and air dried. Fruit harvested at MG (named as ‘FHM’) were stored in the dark and analyzed when they reached Turning ‘T’ after storage at (i) 20°C (named as ‘20T’); (ii) 12.5°C (named as ‘12.5T’), and (iii) 5°C for two weeks followed by rewarming at 20°C (named as ‘5T’). The control group is the fresh harvested Turning fruit (‘FHT’). MG fruit were also analyzed after storage at 5°C for 2 weeks (‘5M’). Three biological replicates, each consisting of a pool of six randomly selected fruit pericarps, were sampled for whole-genome bisulfite sequencing, RNASeq, carotenoids, and ABA assays.

### Whole genome bisulfite sequencing

#### Genomic DNA extraction

Genomic DNA was isolated using the Qiagen® DNeasy Plant Mini Kit. Due to the high carbohydrates of ripening tomato fruit, the procedures were modified according to the manufacturer’s protocol to increase DNA yields and quality. The extraction for each sample was started with a duplicate sample material, and one extraction of 100 mg frozen fresh fruit powder were added into the buffer AP1 and P3 followed by QIAshredder columns, respectively. The flowthrough from the duplicate extractions was pooled together, and after adding AW1, all mixtures were loaded into one DNeasy Mini spin column. In the final elution, the AE buffer was preheated at 65°C and incubated for 30 min for the best elution efficiency. The isolated DNA was further purified using the DNA Clean & Concentrator-5 (Zymo Research Corp., Irvine, CA, USA). The quality of DNA was assessed on the 0.8% (w/v) agarose gel, a NanoDrop™ 1000 Spectrophotometer (Thermo Scientific, MA, USA) and a Bioanalyzer (Agilent, Santa Clara, CA, USA).

#### Methyl-Seq library preparation and sequencing

The bisulfite conversion of sonicated genomic DNA fragments was carried out based on the instructions provided in the EZ DNA-methylation lightning Kit (Zymo Research Corp., Irvine, CA, USA). The libraries were made using the Accel-NGS Methyl-Seq DNA library kit (SWIFT Biosciences, Ann Arbor, MI, USA) and quality checked using the Bioanalyzer. The libraries were sequenced using the NovaSeq PE 150 at the UC Davis Genome Center DNA Technologies & Expression Analysis Core.

#### Data processing

The sequencing reads were first quality checked on FastQC [92], and all libraries passed quality control requirements, after adaptor trimming using Trimmomactic [93]. The bisulfite conversion rates were calculated by aligning reads to the unmethylation chloroplast genome, and the conversion rates for all libraries were more than 97% [94]. The trimmed reads were aligned to the tomato genome assembly SL4.0 (Sol Genomic Network) using Bismark [95]. The multialigned reads were deduplicated to remove PCR bias. Methylation extraction was conducted to calculate the methylated status of each sequenced cytosine and extracted by CpG, CHH, and CHG contexts respectively. The visualization of the DNA methylation status and correlation between each library were performed in SeqMonk (https://www.bioinformatics.babraham.ac.uk/projects/seqmonk/). The final Bismark output text files were imported to R (R Core Team, 2020). The DMRs and DMGs (*P* < 0.05) were extracted using MethylKit [96] and were annotated using the Genomation package [97]. The DMRs were defined by a threshold of *P* < 0.05, the difference of the methylation percentage > 10, using a 200-bp sliding window. The DMGs were defined as having DMRs around the gene body or 3 kb upstream promoter regions [98].

### RNASeq library preparation and sequencing

#### RNA isolation

Fruit pericarp were frozen by liquid nitrogen and stored at −70°C upon sampling. Total RNA was isolated from around 100 mg fruit powder using a Trizol-based protocol. RNA quality and integrity were assessed by NanoDrop™ 1000 Spectrophotometer (Thermo Scientific, MA, USA) and 0.8% (w/v) agarose gel electrophoresis. The mRNA was isolated from total RNA using NEBNext® Poly(A) mRNA Magnetic Isolation Module.

#### 3′ DGE RNASeq library construction and sequencing

The libraries were built using strand-specific mRNA-library prep kits (Amaryllis Nucleics, Oakland, CA). All libraries that passed the quality check conducted by Novogene were pooled into one lane and sequenced by HiSeq PE150. The raw sequencing reads were trimmed for removing adaptors using Trimmomatic [93] and quality checked by FastQC [92]. The reads alignment was processed by STAR [99] based on the tomato reference genome SL4.0 (Sol Genomic Network). Visualization of the aligned reads was performed in SeqMonk. The aligned reads were imported to R and processed by the package FeatureCounts [100] to obtain the read count of each gene. Data normalization and clustering were performed before extracting DEGs by EdgeR [101]. The threshold of DEGs is log_2_ fold change >1 and adjusted *P* < 0.01. The input of the GO terms was downloaded using the BioMart tool at Ensembl Plants (http://plants.ensembl.org/biomart/martview/) for both DEGs and DMGs annotation. The functional enrichment analyses including Gene ontology by GOseq [102], and KEGG by Gage [103] were conducted.

### Bioinformatics analysis

#### Co-expression network

Gene modules were identified using the WGCNA under the R environment [104], from 15 samples (‘5T’, ‘12.5T’, ‘20T’, ‘FHT’, and ‘5M’, each with three biological replicates) in the RNASeq data. The correlation network analysis included 2255 significant genes identified in at least one comparison between postharvest Turning fruit and ‘FHT’, i.e. ‘5T’ vs. ‘FHT’, ‘12.5 T’ versus ‘FHT’, and ‘20T’ versus ‘FHT’. The power (soft threshold) was determined by the pickSoftThreshold function in the WGCNA package. An unsigned network was constructed using automatic network construction, with minModuleSize of 30 and mergeCutHeight of 0.25. The eigengene expressions were obtained, and Pearson’s correlation coefficient (PCC) represented by *r* was used to calculate the correlation between each module and treatment group. Furthermore, the top 1000 strongest connections, identified as gene pairs with the highest edge weight, were further imported to Cytoscape (version 3.9.1) [105] for network visualization.

#### Hub genes

Hub genes in each module were identified through a multicriteria approach. First, genes with the top 10% intramodular connectivity were selected. The intramodular connectivity was calculated using the function intramodularConnectivity in the WGCNA. Second, the selected genes were further filtered for the absolute geneModuleMembership (KME) value greater than 0.9, where the KME value was calculated by signedKME in the WGCNA package. The filtered genes were then combined with the top 1000 strongest connections identified in section above to find those that overlapped. The overlapped genes were identified as the hub genes that are strongly associated with and highly connected within candidate modules.

#### Gene ontology visualization using GoFigure

GoFigure [37], a Python package, was used for GO visualization. The GO categories and the associated overrepresented *p*-values for each module were imported into the program to create the plots.

#### Enrichment analysis using DAVID

The Database for Annotation, Visualization and Integrated Discovery (DAVID) [36, 106] as used for functional annotation for DEGs, DMGs, and genes in each cluster identified in WGCNA. Gene IDs input into the DAVID were converted to SL3.0 to be mapped to DAVID IDs. Functional annotation terms with an adjusted *P*-value less than 0.05 and functional annotation clusters with an enrichment score greater than 1.3 were considered significant.

#### Transcriptomic analysis by pathways and expression heatmaps

The Log_2_ (Counts per million-CPM) values from RNASeq data were used as input for each pathway analysis. Statistical significance was determined using Tukey’s multigroup tests among all four Turning groups, with asterisks and red lines added indicating DEGs at *P* < 0.05. The DEGs were decided without filtering by gene expression fold-change. This method was applied across the gene expression heatmaps of the carotenoids, ABA, ethylene, photosynthesis, and ripening TFs in this work, and, those in the supplementary files. For the DEGs, a zoomed color scale was used to adjust the colors in the expression heatmap within a narrower range (−1 to 1). This enables better visualization of subtle changes in DEGs’ expression.

#### Correlations between gene associated DNA methylation regions and gene expression levels

The correlation between gene expression and DNA methylation levels was calculated for each DMR determined in three methylation contexts, i.e. CG, CHG, and CHH. For each DMR, the RNASeq data with three biological replicates were used as the gene expression levels, and the average DNA methylation percentage across all contexts was used as the DNA methylation levels. Correlations were calculated separately if there were multiple DNA methylation sliding windows identified for one gene. The PCC represented by *r* and its *P*-values were calculated to indicate the strength of correlation.

For genes in specific pathways, the correlation between their gene expression and DNA methylation levels was examined. The DNA methylation levels were based on the regions surrounding the gene, including the 3 kb upstream and gene coding regions. The correlation was indicated by *r*, and statistical test indicated by *p*-values were summarized in tables.

### Fruit carotenoids

Carotenoids extractions and assay were done as previously described [107] with some modifications. Frozen tomato tissue (0.2–0.4 g) was extracted with 20 mL HEA (2:1:1 hexane: ethanol: acetone, v/v/v) containing 0.1% (w/v) butylhydroxytoluene. The extracted carotenoids were covered with aluminum foil to avoid light exposure. The extraction was repeated to collect all supernatants after centrifugation until the tomato tissue was colorless. The homogenized extract was incubated for 15 min in the dark at room temperature, and 15 ml distilled water was added, and the extract was incubated further for 15 min. The organic phase was separated and evaporated under high pressure N_2_ until dry. Carotenoids contents were analyzed using high performance liquid chromatography (HPLC; Agilent 1100, Hewlett-Packard-Strasse, Germany). The dried extract was dissolved in 1 mL of the mobile phase (10: 5: 85 dichloromethane: acetonitrile: ethanol, v/v/v) [108] and filtered through a 0.22-μm nylon membrane. The sample (20 μL) was injected into the HPLC equipped with a YMC-C30 reversed-phase column (25 mm × 4.6 mm, 5 μm, YMC Co., Kyoto, Japan). The flow rate was 1 mL/min at ambient temperature (25°C), and the absorption of each compound was detected with a UV–Vis detector. Absorption spectra for the main peaks were 285 nm for phytofluene and 450 nm for lycopene, β-carotene, and lutein. A chromatographic run lasted 65 min. Each carotenoid was identified by the retention time compared with the external standard. Phytofluene standards were purchased from CaroteNature GmbH (Lupsingen, Switzerland). Lycopene (9879), β-carotene (22040), and lutein (07168) standards were purchased from Sigma-Aldrich, USA.

### Fruit ABA extraction and ELISA–antibody kit analysis

The ABA extraction methods were modified from a previous study [26]. Approximately 50–100 mg of frozen tomato tissues were ground in liquid nitrogen and used for the extraction. One milliliter of the extraction buffer (80% methanol (methanol: water: acetic acid (80:19:1, v/v/v) with 100 mg/L butylated hydroxytoluene (BHT)) was added in each sample, and the incubation was conducted at 4°C in the dark. After 24 h, the supernatant and pellet were separated by centrifuging, and the incubation was repeated using another 1 mL extraction buffer for an additional hour. All supernatants were collected and dried in a speed vac. The dry pellet was dissolved in 99% methanol (methanol: acetic acid (99:1, v/v) with 100 mg/L BHT. The dissolved pellet was added with 900 μl 1% (v/v) acetic acid, loading into the Sep-pak C18 reverse phase columns (Waters, USA). The column was washed with 3 ml of 20% (v/v) methanol following elution by 3 ml of 80% methanol (methanol: water: acetic acid (80:19:1, v/v/v) with 100 mg/l BHT. The eluted samples were dried, and the pellet was dissolved with 50 μl methanol and 450 μl tris-buffered saline (TBS) buffer. The extracts were diluted 20-fold using TBS buffer before the Phytodetek® ELISA-plant ABA kit assay (Agdia, Inc., Elkhart, IN).

### Fruit difference of absorbance (DA) index and color assay

A DA meter® (TR Turoni, Italy) was used for the nondestructive assessment of fruit chlorophyll content, while a colorimeter (Konica Minolta, Tokyo, Japan) was used for measuring objective color. The color was used as the determinant for fruit developmental stage in this study. Each fruit was assessed twice at the equatorial regions of the skin according to Albornoz *et al*. [14]. At least 20 tomato fruit were measured in each treatment group. The *I*_DA_ is the difference in absorbance between 670 and 720 nm, and chlorophyll *a*, the main chlorophyll in ripening tomato fruit, peaks at 660 nm [109]. *I*_DA_ is highly correlated with fruit skin color and chlorophyll contents in tomato [110], and lower *I*_DA_ is recorded as the fruit ripens.

### Fruit postharvest gas analysis: ethylene and respiration rates

Tomato fruit at the MG stage were harvested in the morning and stored under different temperatures. The gas assays were performed daily at a similar time. Around 100 g of fruit were pooled in one jar as one biological replicate. Six biological replicates, each with at least two repeated assays (technical replicates), were included. The fruit were placed in a sealed 450 ml glass jar for 30–60 min each day, and gas was extracted for assaying ethylene and CO_2_. Ethylene was measured by a gas chromatograph, and carbon dioxide was assayed by a CO_2_ analyzer [14].

### Validation of the RNASeq identified DEGs using RT-qPCR

Fruit harvesting and postharvest treatments were repeated to neutralize pre-harvest environmental factors affecting the fruit transcriptome. Tomato plants were grown in the greenhouse at UC Davis, CA in 2023. Postharvest treatments were performed on fruit randomly harvested over 50 plants. Six fruits were randomly selected and pooled together to form one biological replicate. Three biological replicates and four technical replicates were included. Fruit pericarp samples were frozen into liquid nitrogen and stored at −70°C upon sampling. Total RNA was isolated from 100 mg fruit powder using a Trizol-based protocol. RNA quality and integrity were assessed by NanoDrop™ 1000 Spectrophotometer (Thermo Scientific, MA, USA) and 0.8% (w/v) agarose gel electrophoresis. cDNA libraries were reverse transcribed, and RT-qPCR was performed according to our previous study [8]. The *SlFRG27* (*Solyc06g007510*) was the internal control reference gene for all tested genes [111]. The ‘FHT’ was used as the control to compare with each postharvest treatment.

## Supplementary Material

Web_Material_uhae095

## Data Availability

The RNASeq and WGBS sequencing data has been uploaded to Sequence Read Archive (SRA) of NCBI, and the BioProject ID PRJNA1026769 https://www.ncbi.nlm.nih.gov/bioproject/PRJNA1026769
