# Supplementary material for: Integrative analysis of the methylome and transcriptome of tomato fruit (Solanum lycopersicum L.) induced by postharvest handling
Source: Hortic Res. 2024 Mar 25;11(6):uhae095. doi: 10.1093/hr/uhae095 (PMC11151332; doi:10.1093/hr/uhae095)
Supplement: Web_Material_uhae095 [file web_material_uhae095.zip › Zhou_et al. 2023_Supplementary_0208.pdf]

SUPPLEMENTARY DATA

Integrative analysis of the methylome and transcriptome of tomato fruit (*Solanum lycopersicum* L.) induced by postharvest handling

Jiaqi Zhou[1], Sitian Zhou[2], Bixuan Chen[1], Kamonwan Sangsoy[3], Kietsuda Luengwilai[3], Karin Albornoz[4], Diane M. Beckles[1*]

[1]Department of Plant Sciences, University of California, Davis, CA, USA;

[2]Department of Biostatistics, School of Public Health, Columbia University, New York, New York 10032, USA;

[3]Department of Horticulture, Faculty of Agriculture at Kamphaeng Saen, Kasetsart University, Kamphaeng Saen Campus, Nakhon Pathom 73140, Thailand;

[4]Department of Food, Nutrition, and Packaging Sciences, Coastal Research and Education Center, Clemson University, Charleston, SC, USA.

[*]Corresponding author. Email: dmbeckles@ucdavis.edu

List of tables and figures in supplementary information

Supplementary Table S1. Statistics of Bisulfite Seq and RNASeq

Supplementary Table S2. Context-specific differentially methylated regions (DMRs) and differentially methylated genes (DMGs).

Supplementary Table S3. Summary of DMGs in all contexts

Supplementary Table S4. DMGs enrichment analysis by DAVID

Supplementary Table S5. Differential expressed genes (DEGs) summary

Supplementary Table S6. DEGs gene ontology enrichment analysis

Supplementary Table S7. DEGs enrichment analysis by DAVID

Supplementary Table S8. Hub genes from WGCNA

Supplementary Table S9. WGCNA hub genes enrichment analysis by DAVID

Supplementary Table S10. Summary of context specific DMGs and the correlation between gene expression and DNA methylation

Supplementary Table S11. Correlative analysis between differential expressed genes (DEGs) in RNASeq and their DNA methylation

Supplementary Table S12. Gene lists in quality related pathways

Supplementary Table S13. Summary of genes of quality related pathways that with significant correlation between DNA methylation and gene expression

Supplementary Table S14. Ethylene and CO_2 assay total production rates and statistical test

Supplementary Table S15. DEGs associated with DMRs

Supplementary Figure S1. Context-specific DNA methylation percentages in tomato

Supplementary Figure S2. Circular plot of the DNA global methylation difference in the CpG context

Supplementary Figure S3. Fruit methylome context-specific differentially methylated regions (DMRs)

Supplementary Figure S4. Hierarchical clustering of the most variable genes in the transcriptome

Supplementary Figure S5. Weighted gene co-expression network analysis (WGCNA)

Supplementary Figure S6. The heatmap of eigengene expression of WGCNA

Supplementary Figure S7. ME turquoise genes in top 1000 connectivity identified in each ME

Supplementary Figure S8. ME blue genes in top 1000 connectivity identified in each ME

Supplementary Figure S9. ME brown genes in top 1000 connectivity identified in each ME

Supplementary Figure S10. ME green genes in top 1000 connectivity identified in each ME. The color of each node represents the node degree

Supplementary Figure S11. ME yellow genes in top 1000 connectivity identified in each ME. The color of each node represents the node degree

Supplementary Figure S12. ME grey genes in top 1000 connectivity identified in each ME. The color of each node represents the node degree

Supplementary Figure S13. GO figure output of the WGCNA

Supplementary Figure S14. DAVID enrichment analysis of genes in WGCNA clusters

Supplementary Figure S15. DAVID enrichment analysis of differential methylated genes (DMGs) in the ‘12.5T’

Supplementary Figure S16. Transcriptomic analysis by KEGG annotation

Supplementary Figure S17. Transcriptomic analysis in fruit cell wall pathway

Supplementary Figure S18. Transcriptomic analysis in DNA methylation and histone related pathways

Supplementary Figure S19. Transcriptomic analysis in auxin/IAA related genes.

Supplementary Figure S20. Ethylene production fitting curves

Supplementary Figure S21. Postharvest green fruit DA index

Supplementary Figure S22. Photosynthetic genes with correlation between gene expression and DA index

Supplementary Figure S23. RT-qPCR validation of the selected DEGs from RNASeq

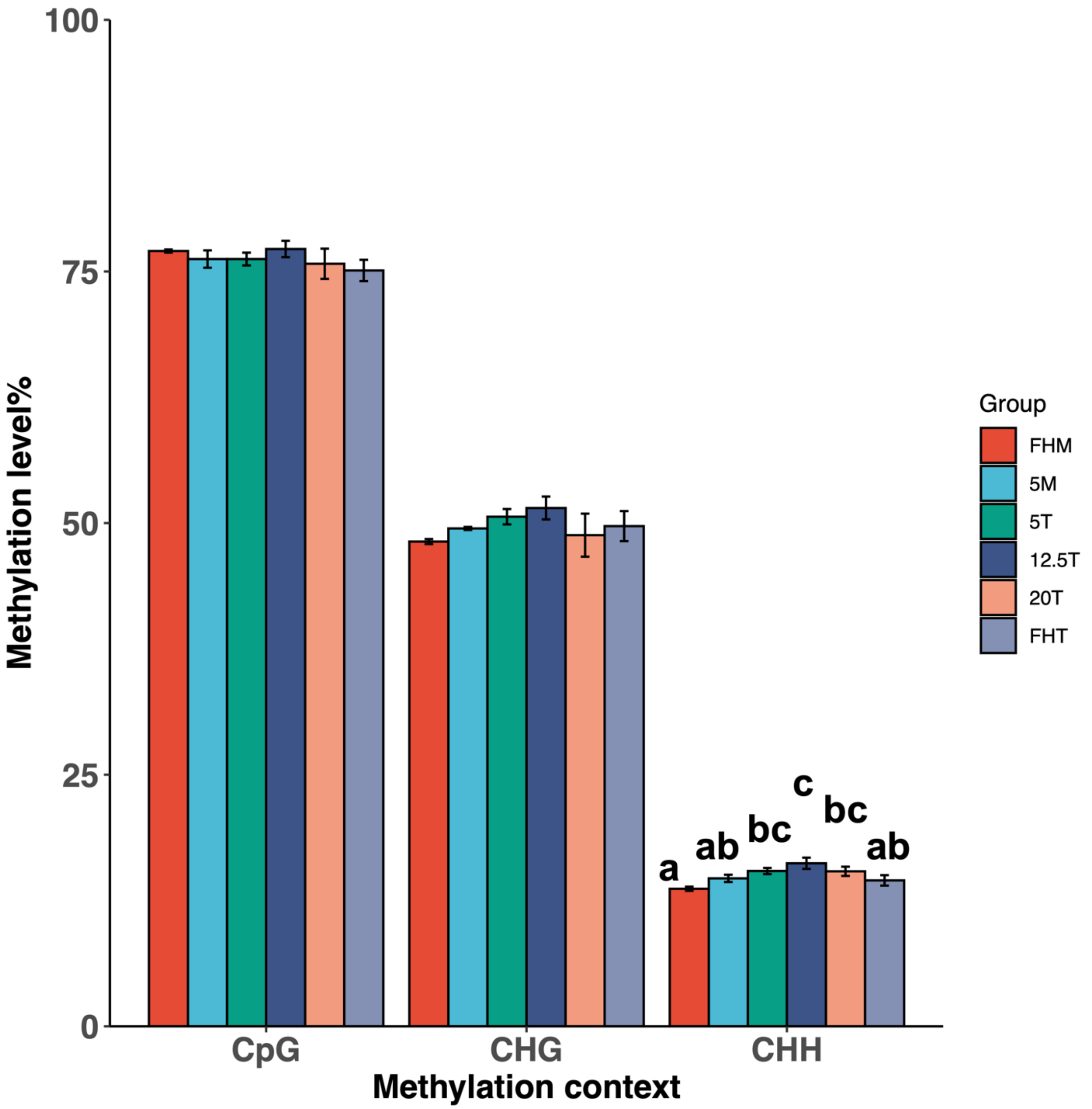

Figure S1 Context-specific DNA methylation percentage in tomato. Genome methylation in tomato fruit at ‘Mature green’ (M) and ‘Turning’ (T) after fresh-harvesting (FH) or storage at different temperatures indicated. Methylation of the ‘12.5T’ fruit was significantly higher than ‘FHM’ fruit ($p < 0.05$) in the CHH context.

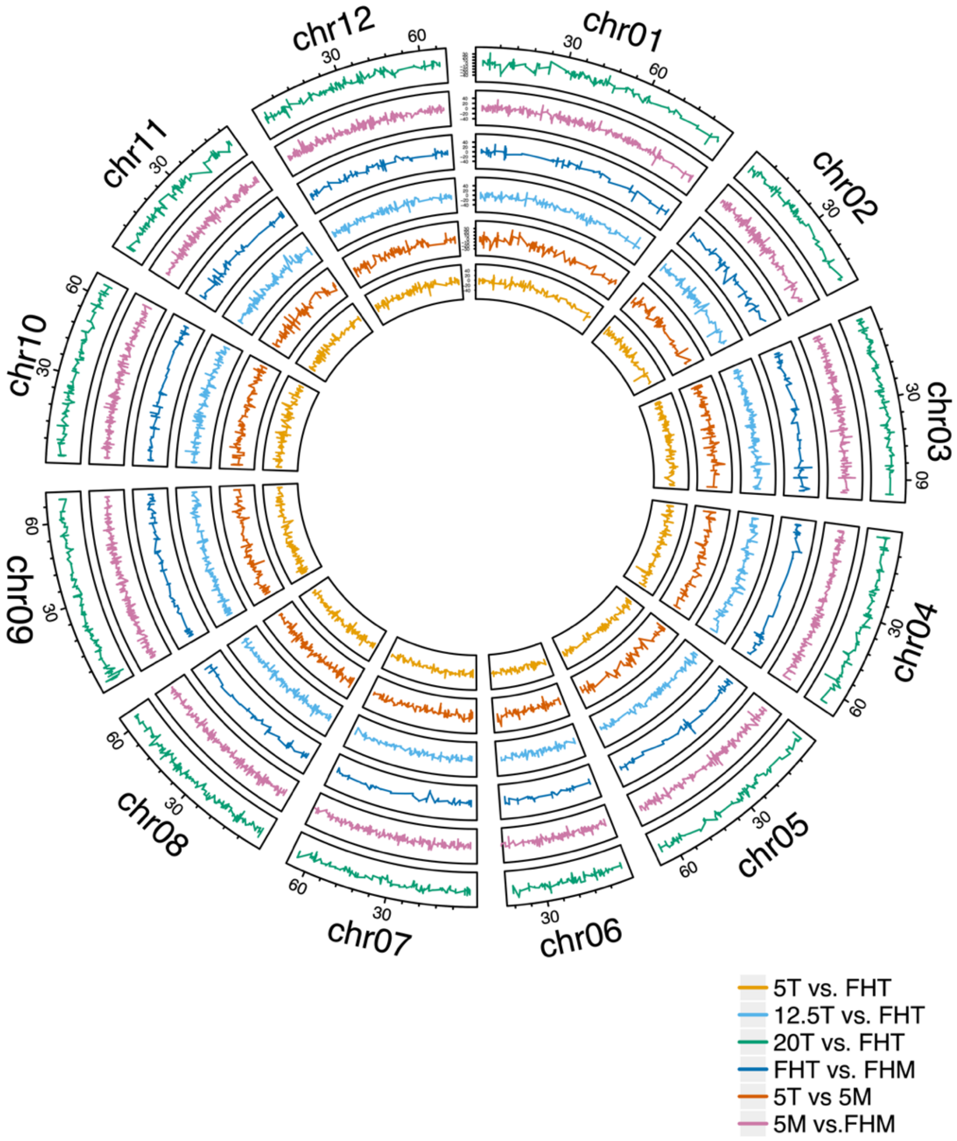

Figure S2 Circular plot of the DNA global methylation difference in CpG context. For each sector, the x-axis represents the methylated cytosine position (in Mb) in the chromosome, and the y-axis represents the methylation difference.

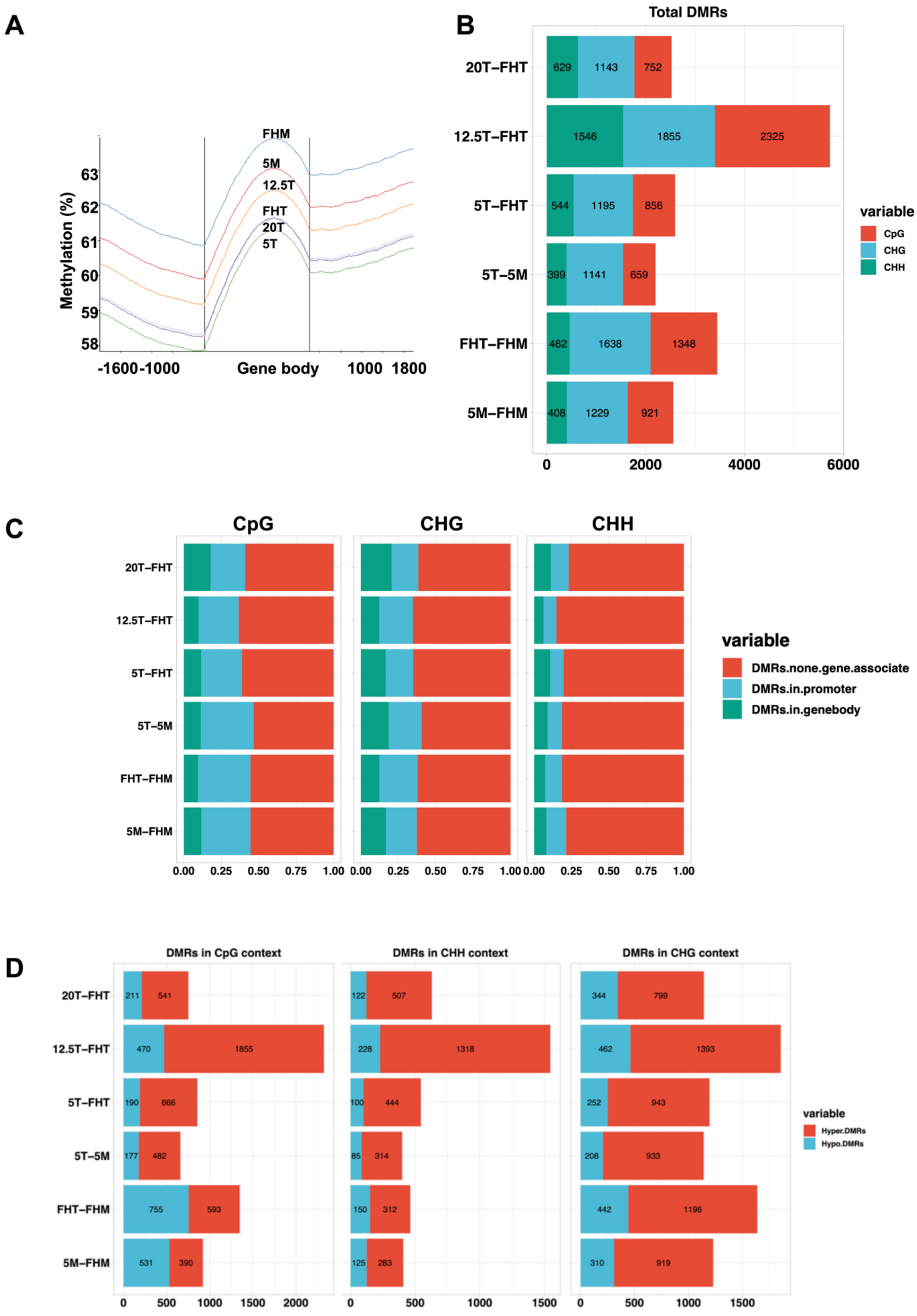
A
Methylation (%)
63
62
61
60
59
58
FHM
5M
12.5T
FHT
20T
5T
-1600-1000
Gene body
1000 1800
B
Total DMRs
20T–FHT
629 1143 752
12.5T–FHT
1546 1855 2325
5T–FHT
544 1195 856
5T–5M
399 1141 659
FHT–FHM
462 1638 1348
5M–FHM
408 1229 921
0 2000 4000 6000
variable
CpG
CHG
CHH
C
CpG
CHG
CHH
20T–FHT
12.5T–FHT
5T–FHT
5T–5M
FHT–FHM
5M–FHM
0.00 0.25 0.50 0.75 1.00
variable
DMRs.none.gene.associate
DMRs.in.promoter
DMRs.in.genebody
D
DMRs in CpG context
DMRs in CHH context
DMRs in CHG context
20T–FHT
211 541
122 507
344 799
12.5T–FHT
470 1855
228 1318
462 1393
5T–FHT
190 666
100 444
252 943
5T–5M
177 482
85 314
208 933
FHT–FHM
755 593
150 312
442 1196
5M–FHM
531 390
125 283
310 919
0 500 1000 1500 2000
0 500 1000 1500
0 500 1000 1500
variable
Hyper.DMRs
Hypo.DMRs

Figure S3 Fruit methylome context-specific differentially methylated regions (DMRs). **(A)** Methylation in the genic regions shown as a change in methylation percentage using a 200C (Cytosine) sliding window. **(B)** Total numbers of DMRs in each comparison at CG, CHG and CHH contexts. **(C)** The proportion of DMRs associated with the gene regions in each context. **(D)** The number of DMRs in pair-wise comparison that are hypo and hyper-methylated, in CpG, CHH and CHG contexts respectively.

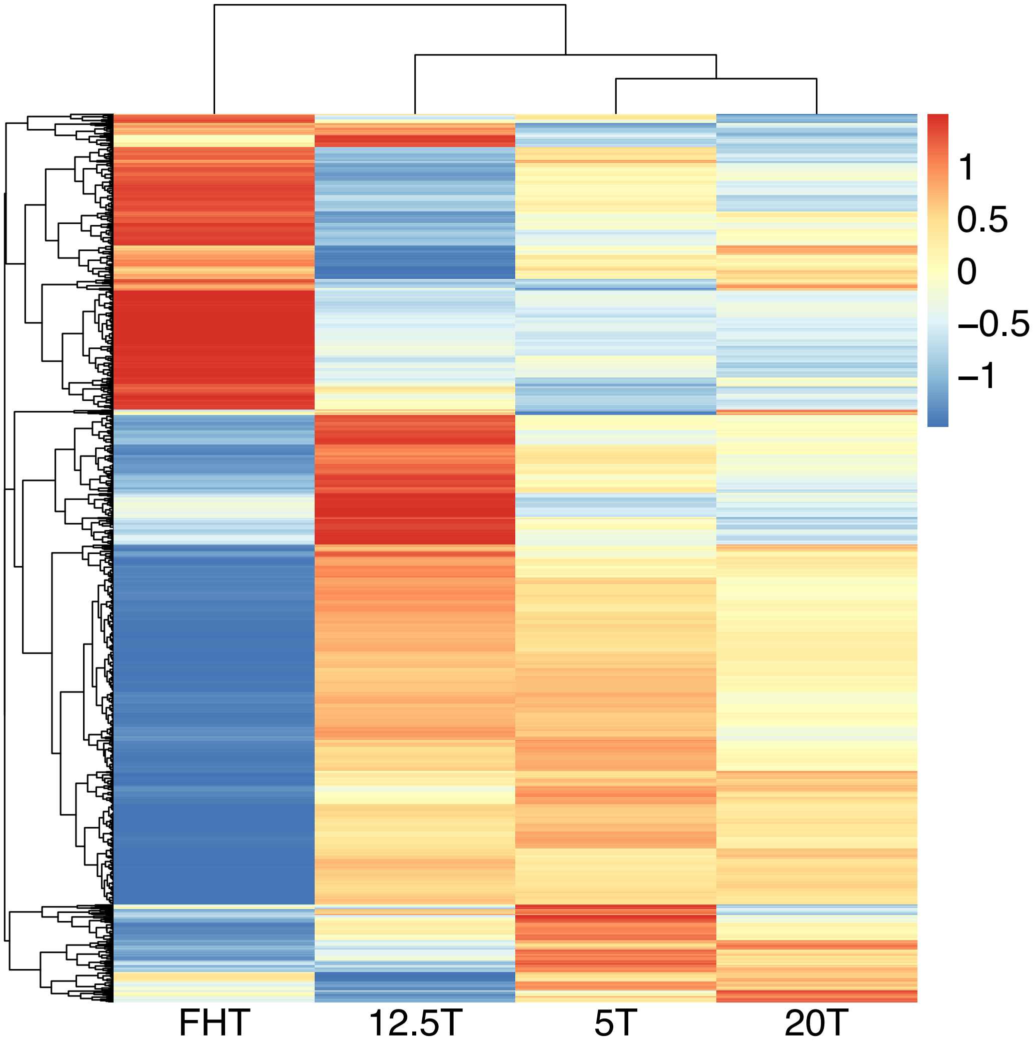

Figure S4 Hierarchical clustering and heatmap were drawn for the most variable 1000 genes based on their expression levels in the RNASeq data. Genes in red and blue represent highly- and lowly-expressed genes, respectively.

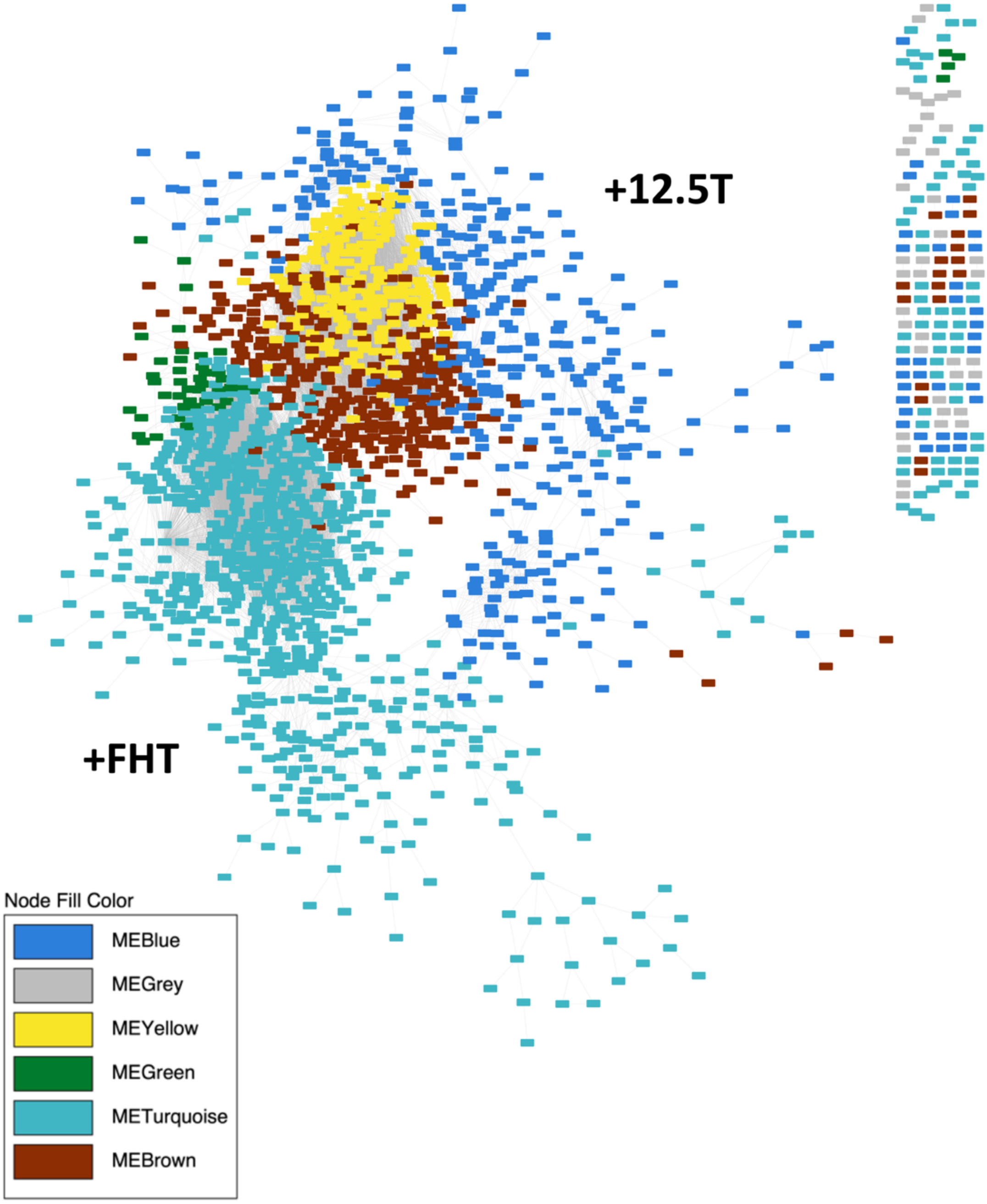

Figure S5 Weighted gene co-expression network analysis (WGCNA). Global view of all clusters explored in this study. The different colors represent different gene clusters according to the figure legend. The ME Turquoise is positively correlated to ‘FHT’ only, and the ME Blue is only positively correlated to ‘12.5T’.

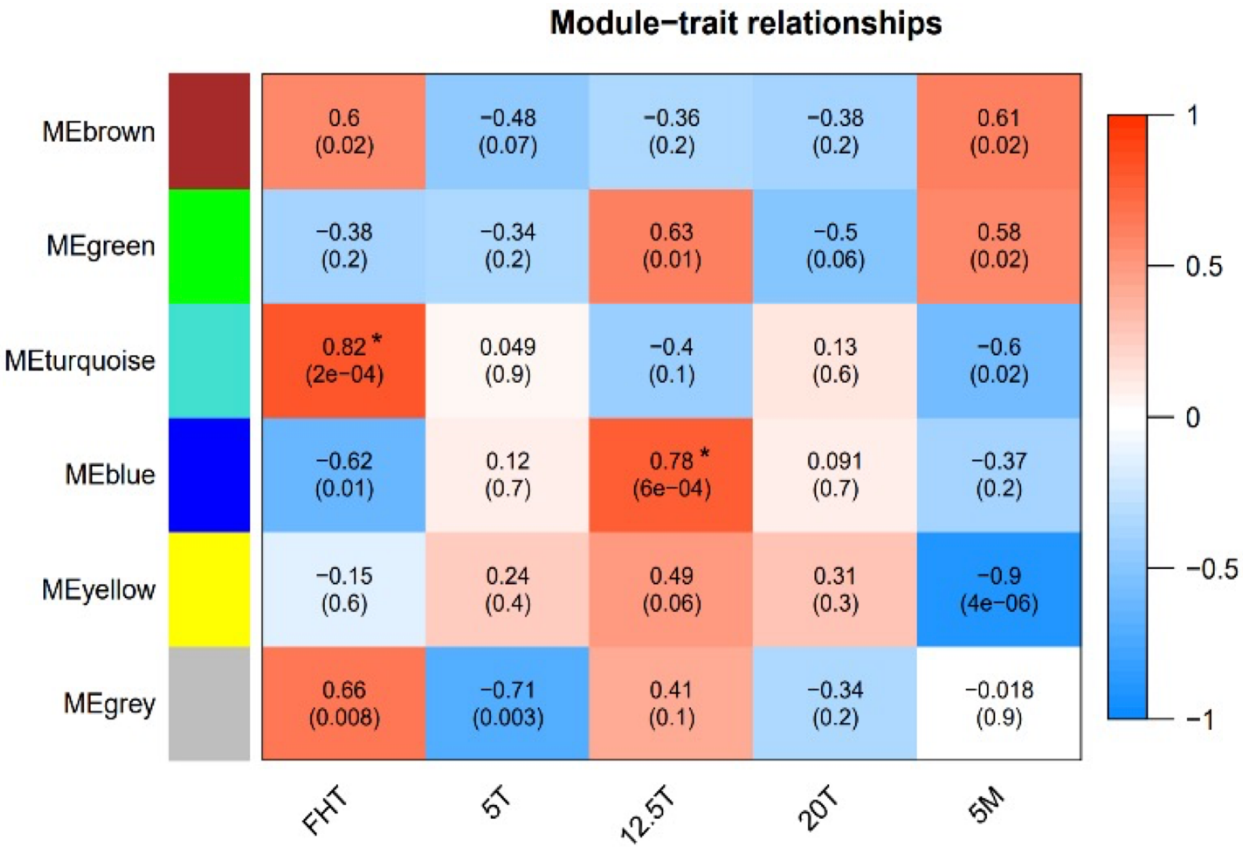

Figure S6 The heatmap of eigengene expression of WGCNA across treatment groups and the module-trait correlations of five samples (‘FHT’, ‘5T’, ‘12.5T’, ‘20T’, and ‘5M’). The top value in each block represents the Pearson’s correlation coefficient (*r*) calculated between the module and the treatment group, and the lower value represents the corresponding *p-value* (* = $p < 0.001$ in at least one turning group).

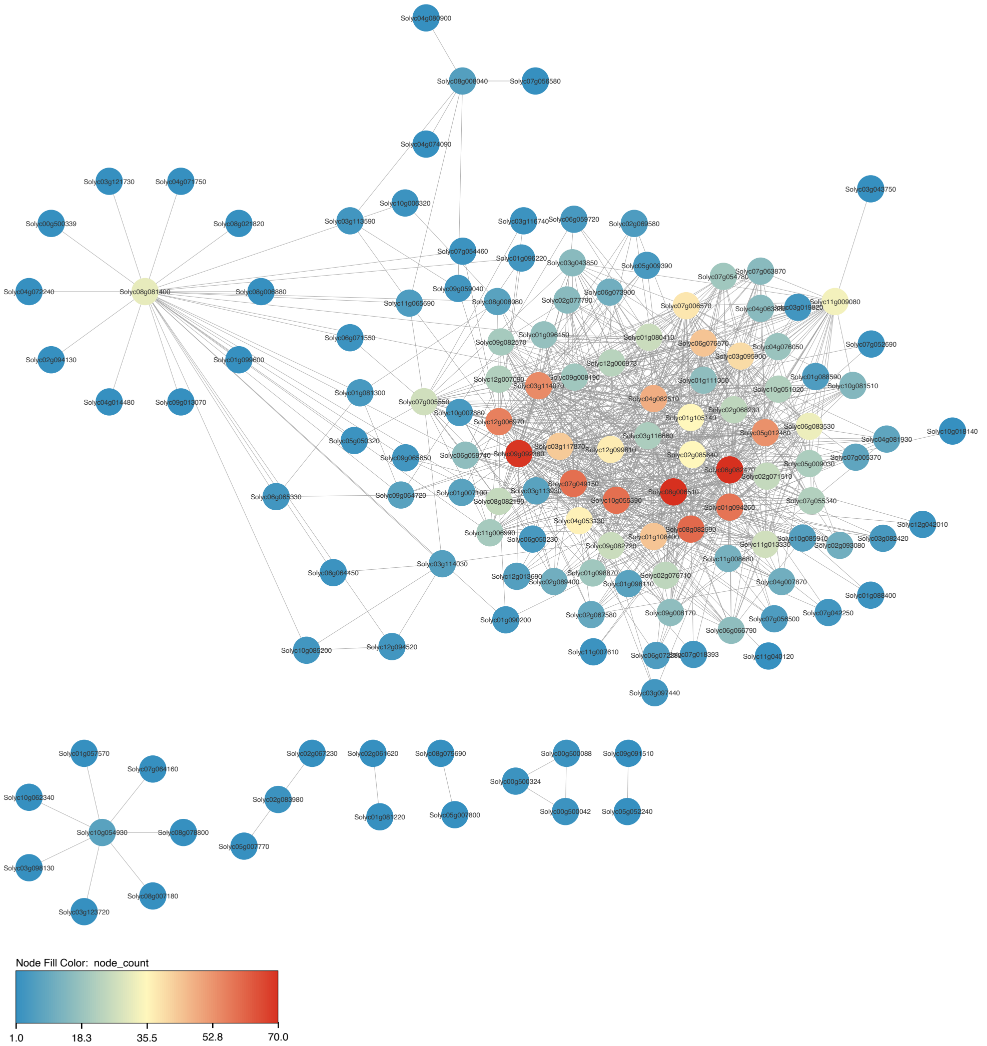

Figure S7 ME turquoise genes in top 1000 connectivity identified in each ME. The color of each node represents the node degree.

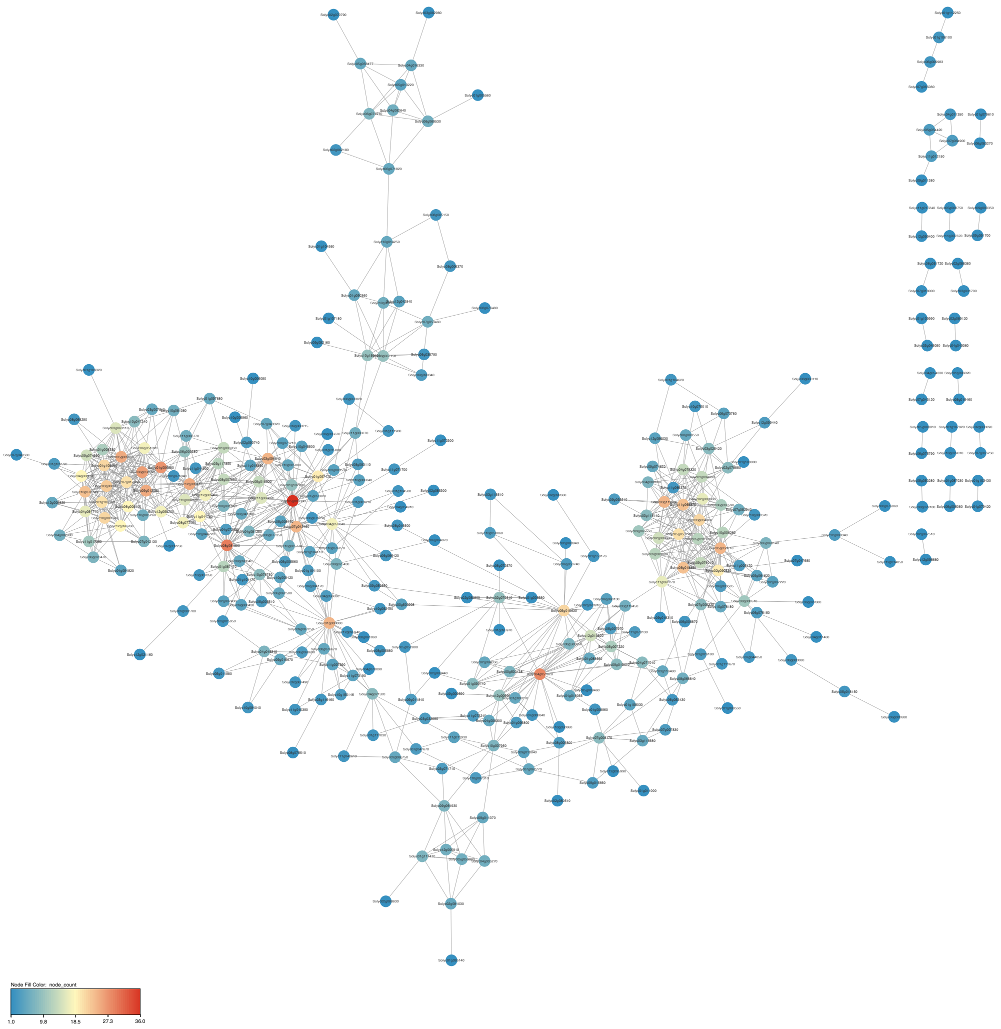

Figure S8 ME blue genes in top 1000 connectivity identified in each ME. The color of each node represents the node degree.

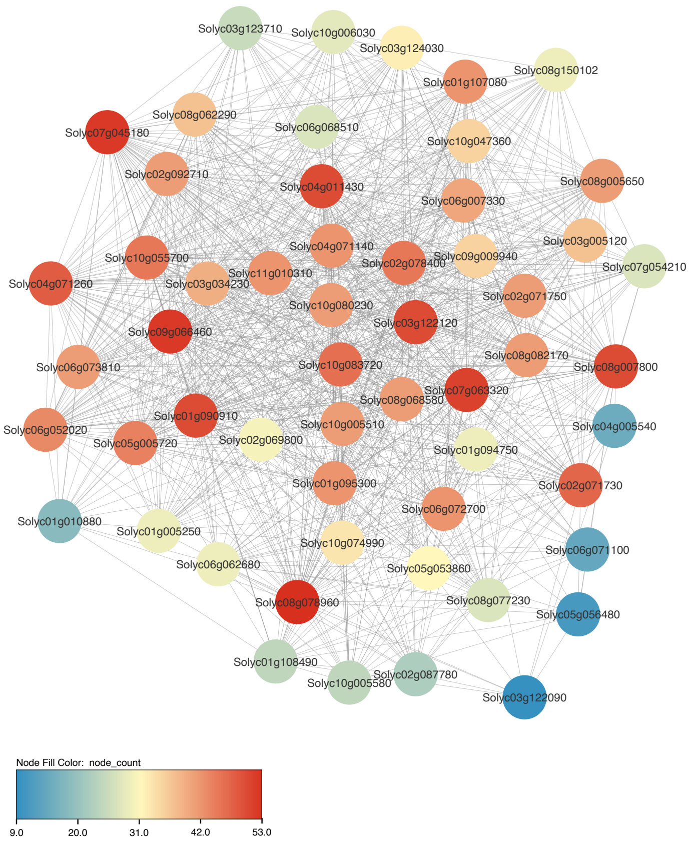

Figure S9 ME brown genes in top 1000 connectivity identified in each ME. The color of each node represents the node degree.

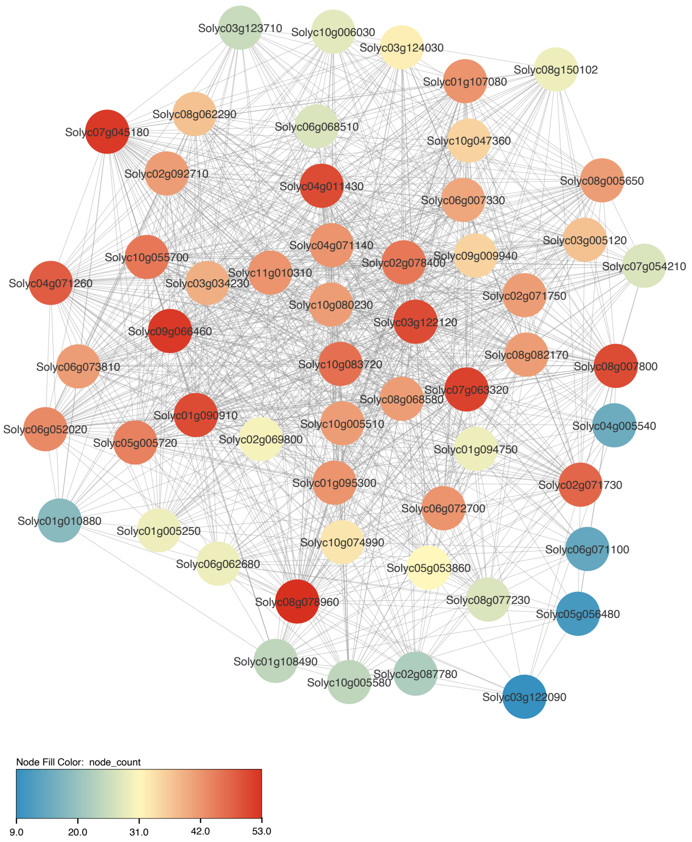

Figure S10 ME green genes in top 1000 connectivity identified in each ME. The color of each node represents the node degree.

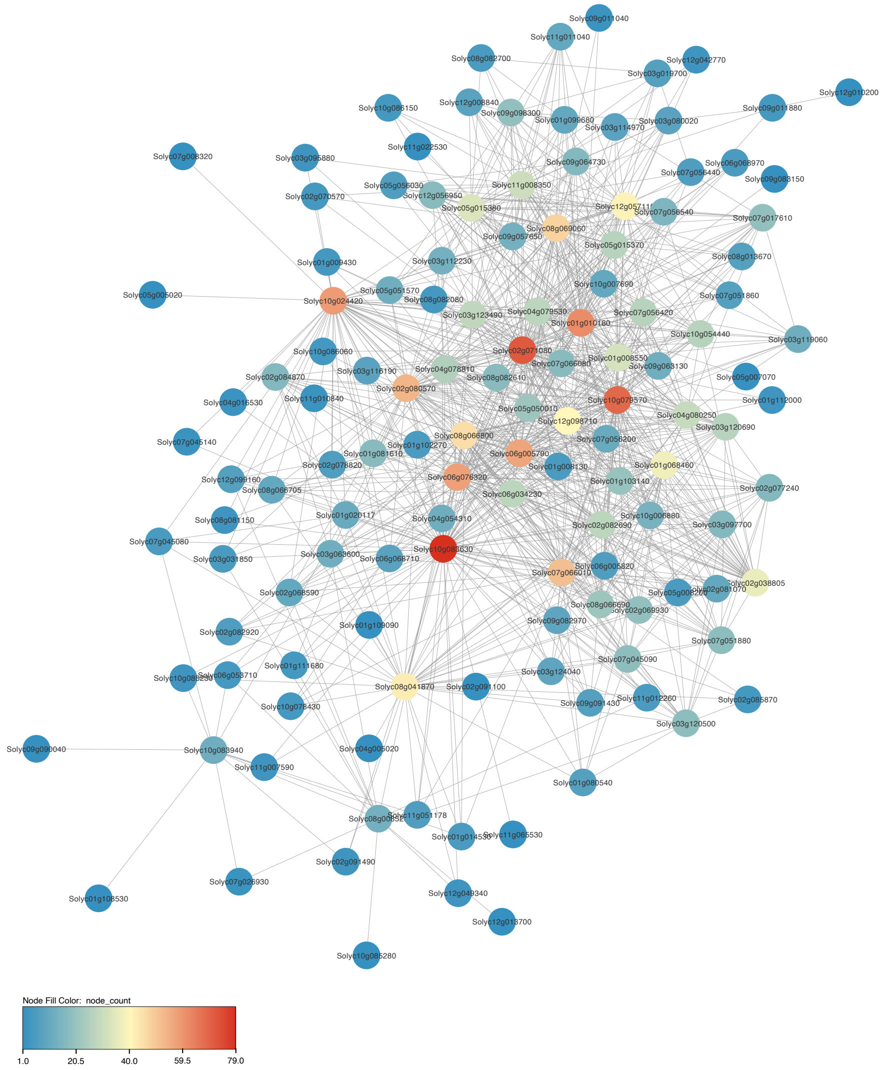

Figure S11 ME yellow genes in top 1000 connectivity identified in each ME. The color of each node represents the node degree.

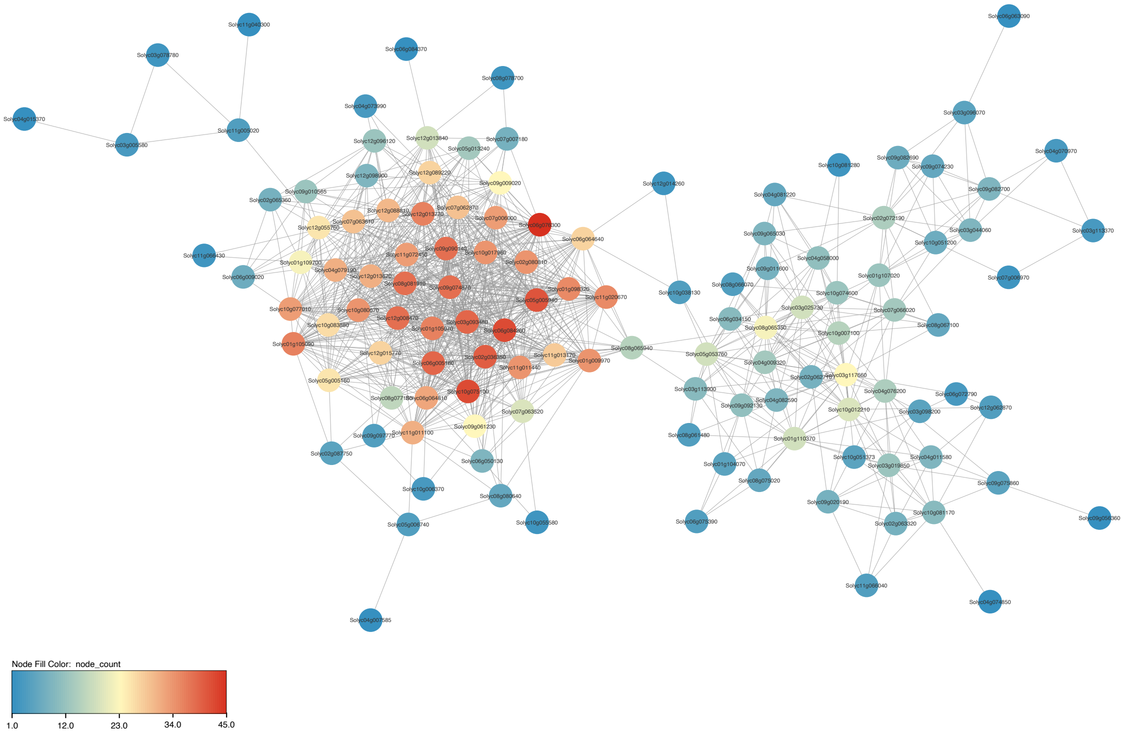

Figure S12 ME grey genes in top 1000 connectivity identified in each ME. The color of each node represents the node degree.

ME turquoise

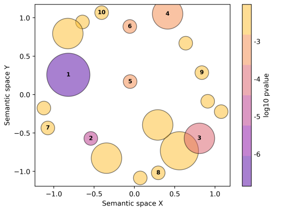

1. response to hydrogen peroxide
2. protein complex oligomerization
3. branched-chain amino acid biosynthetic process
4. response to heat
5. protein folding
6. positive regulation of superoxide dismutase activity
7. pollen maturation
8. Lewis a epitope biosynthetic process
9. polyketide biosynthetic process
10. positive regulation of defense response to insect

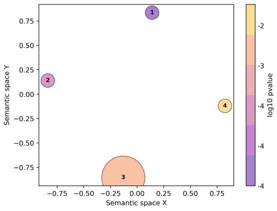

1. cytoplasm
2. chloroplast stroma
3. respiratory chain complex II
4. chloroplast

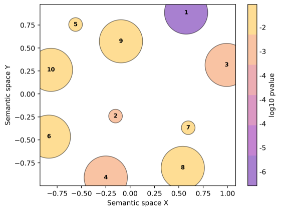

1. protein self-association
2. naringenin-chalcone synthase activity
3. transition metal ion binding
4. dihydroxy-acid dehydratase activity
5. beta-amylase activity
6. serine-tRNA ligase activity
7. oxidoreductase activity
8. 9-cis-epoxycarotenoid dioxygenase activity
9. phosphatidylinositol-4,5-bisphosphate 5-phosphatase activity
10. GTPase activity

ME blue

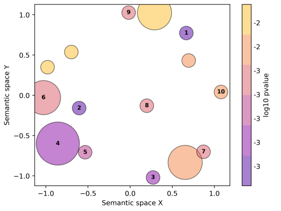

1. fatty acid biosynthetic process
2. regulation of nitrogen utilization
3. response to fungus
4. negative regulation of cell cycle
5. sequestering of actin monomers
6. regulation of auxin mediated signaling pathway
7. detection of ethylene stimulus
8. nucleus organization
9. DNA ligation involved in DNA repair
10. fruit ripening

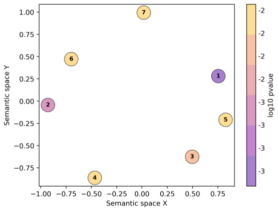

1. chaperonin-containing T-complex
2. nucleolus
3. nuclear pore central transport channel
4. cell cortex
5. acetyl-CoA carboxylase complex
6. chloroplast thylakoid
7. chloroplast stroma

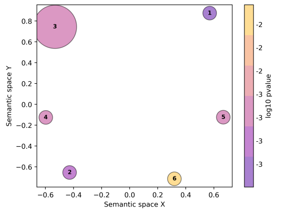

1. actin monomer binding
2. isocitrate dehydrogenase (NADP+) activity
3. DNA ligase activity
4. oxidoreductase activity, acting on the aldehyde or oxo group of donors, disulfide as acceptor
5. 1-aminocyclopropane-1-carboxylate oxidase activity
6. dehydropipecolic acid reductase

ME brown

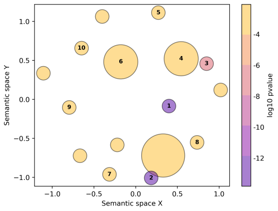

1. photosynthesis
2. translation
3. photosynthetic electron transport in photosystem I
4. photosynthesis, light harvesting in photosystem I
5. response to cytokinin
6. ribosomal small subunit assembly
7. starch biosynthetic process
8. maturation of SSU-rRNA
9. retrograde vesicle-mediated transport, Golgi to endoplasmic reticulum
10. brassinosteroid mediated signaling pathway

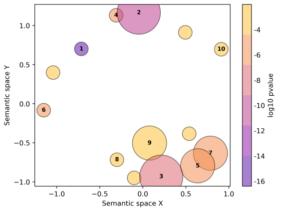

1. chloroplast thylakoid membrane
2. ribosome
3. photosystem II
4. thylakoid
5. cytosolic small ribosomal subunit
6. chloroplast envelope
7. cytosolic large ribosomal subunit
8. photosystem I reaction center
9. photosystem II oxygen evolving complex
10. chloroplast stroma

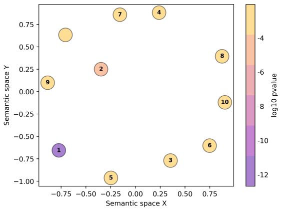

1. structural constituent of ribosome
2. NAD binding
3. catalytic activity
4. AU-rich element binding
5. transporter activity
6. aminoacyl-tRNA hydrolase activity
7. 5S rRNA binding
8. glyceraldehyde-3-phosphate dehydrogenase (NAD+) (phosphorylating) activity
9. chlorophyll binding
10. fructose-bisphosphate aldolase activity

ME green

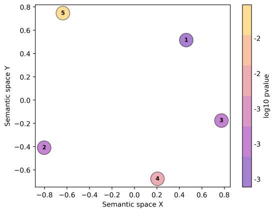

1. cyclic nucleotide metabolic process
2. protein heterotrimerization
3. ethanolamine metabolic process
4. threonine biosynthetic process
5. L-cystine transport

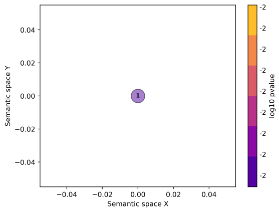

1. chloroplast stroma

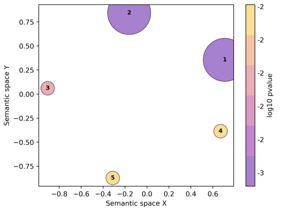

1. aspartate-semialdehyde dehydrogenase activity
2. 2',3'-cyclic-nucleotide 3'-phosphodiesterase activity
3. L-cystine transmembrane transporter activity
4. protochlorophyllide reductase activity
5. NADP binding

ME yellow

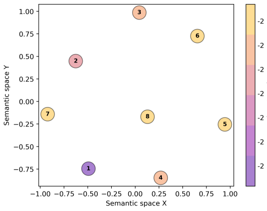

1. photosynthesis
2. starch biosynthetic process
3. regulation of tetrapyrrole metabolic process
4. fatty acid alpha-oxidation
5. UDP-galactose transmembrane transport
6. positive regulation of nuclear-transcribed mRNA poly(A) tail shortening
7. oxidative photosynthetic carbon pathway
8. lysine biosynthetic process via aminoadipic acid

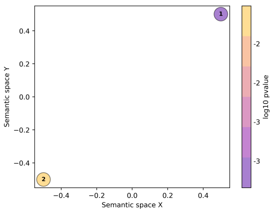

1. photosystem I
2. cytoplasm

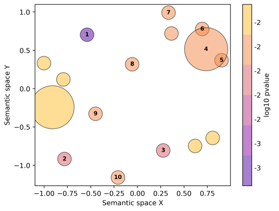

1. catalytic activity
2. calcium-dependent phospholipid binding
3. galactolipase activity
4. 3-oxo-arachidoyl-CoA synthase activity
5. 3-oxo-cerotoyl-CoA synthase activity
6. 3-oxo-lignoceronyl-CoA synthase activity
7. very-long-chain 3-ketoacyl-CoA synthase activity
8. UDP-glucosyltransferase activity
9. magnesium-protoporphyrin IX monomethyl ester (oxidative) cyclase activity
10. UDP-galactose transmembrane transporter activity

ME grey

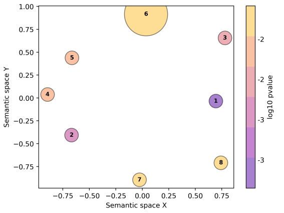

1. cellular oxidant detoxification
2. calcium-mediated signaling
3. carbohydrate transport
4. induced systemic resistance, ethylene mediated signaling pathway
5. positive regulation of transcription by RNA polymerase I
6. protein K69-linked ufmylation
7. microtubule-based process
8. response to cold

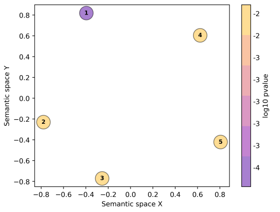

1. chloroplast stroma
2. mitochondrial proton-transporting ATP synthase, stator stalk
3. t-UTP complex
4. plant-type cell wall
5. extracellular exosome

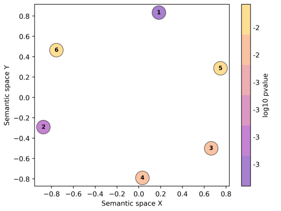

1. intramolecular lyase activity
2. fatty acid binding
3. MAP kinase activity
4. aminomethyltransferase activity
5. catalytic activity
6. copper ion binding

Figure S13 GO figure output of Weighted gene co-expression network analysis (WGCNA) in each ME. The GO output is presented by three categories, i.e., biological progress, cellular component, and molecular function.

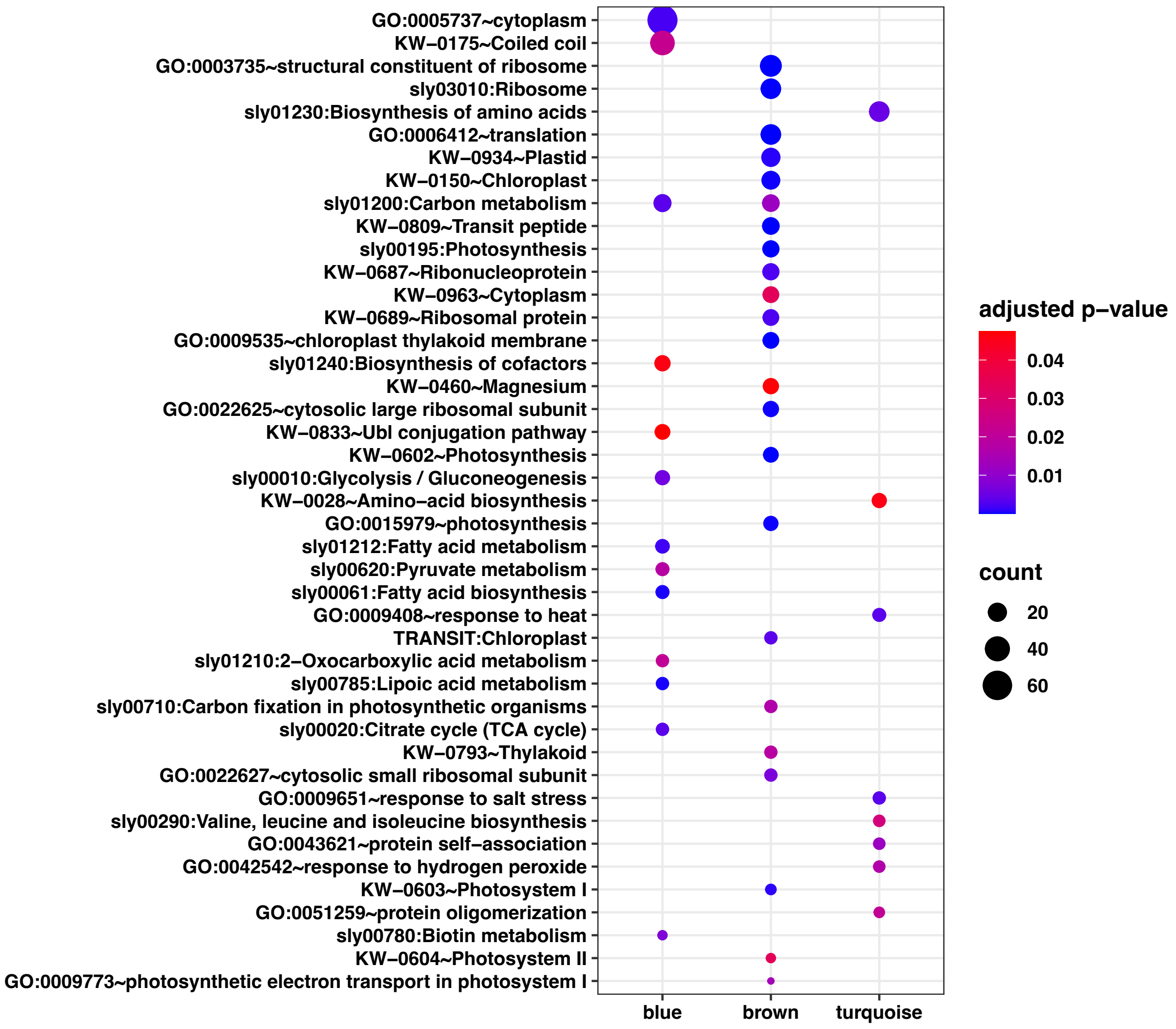

Figure S14 DAVID enrichment analysis of genes in WGCNA clusters. The ME blue, brown and turquoise results were shown in the plot, while for the other clusters not shown, they don’t have significant terms from DAVID (p <0.05).

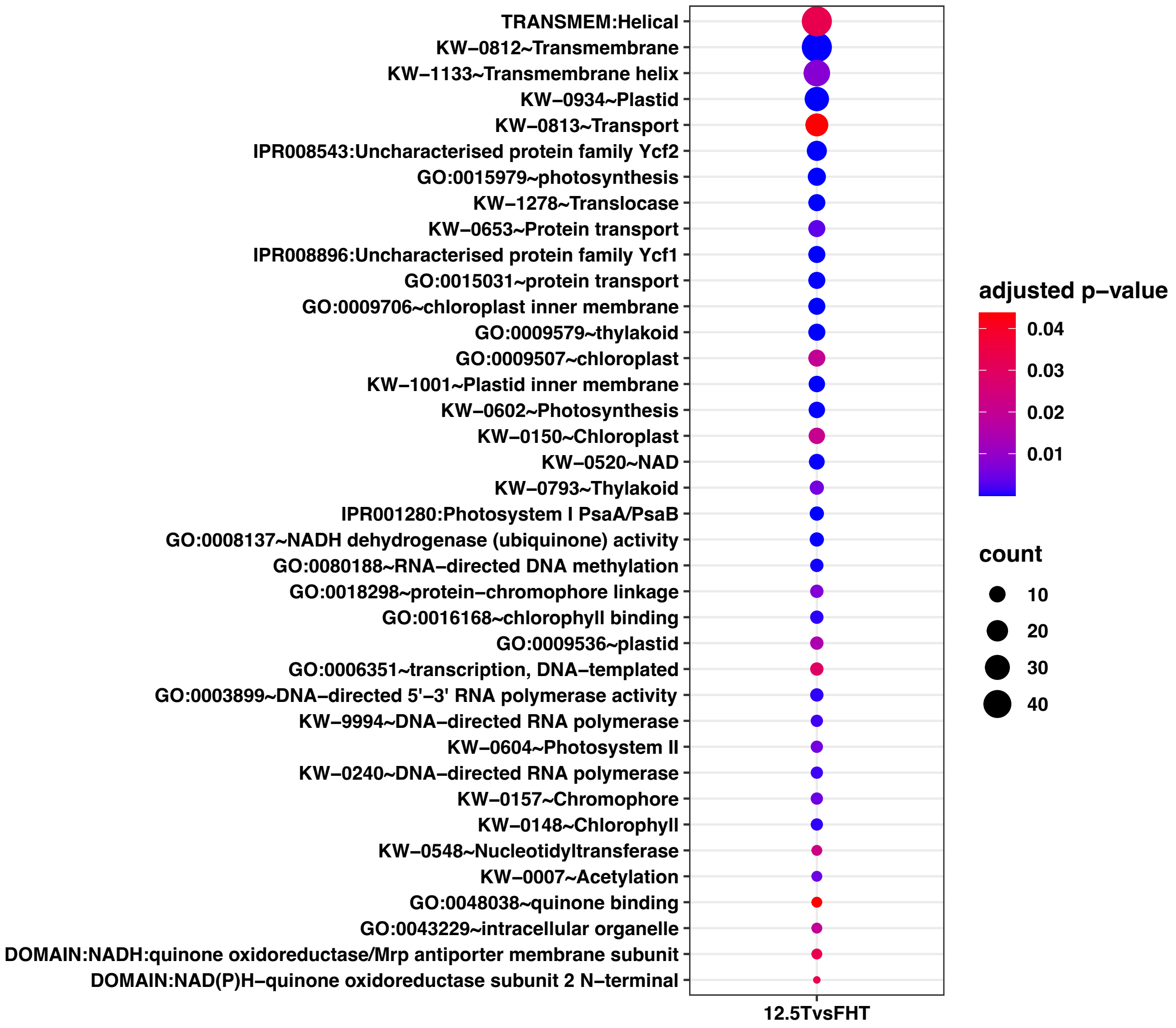

Figure S15 DAVID enrichment analysis of differential methylated genes in '12.5T' (adjusted. *p* < 0.05).

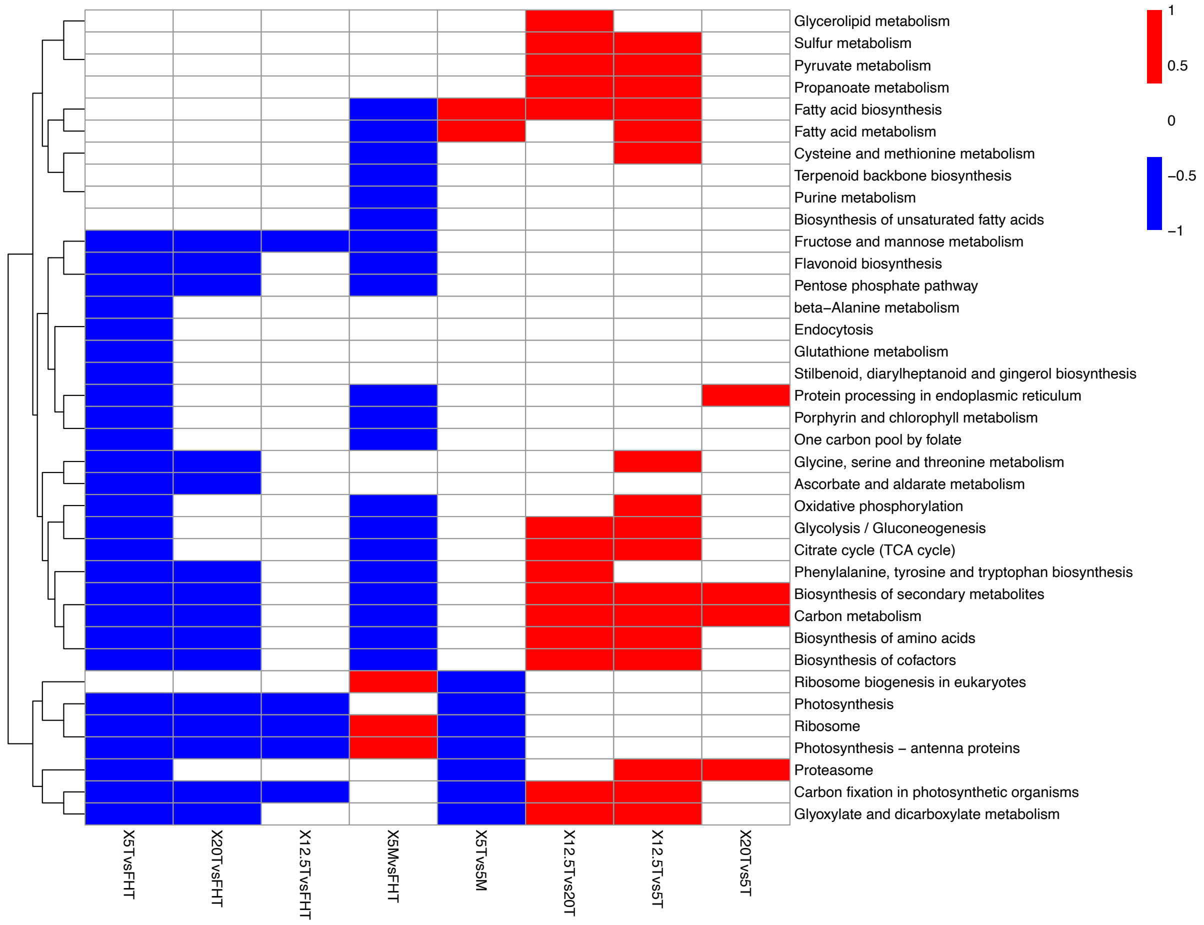

Figure S16 Transcriptomic analysis by KEGG annotation. The boxes in red, blue or white represent upregulation, downregulation, or not significant pathways (FDR< 0.01).

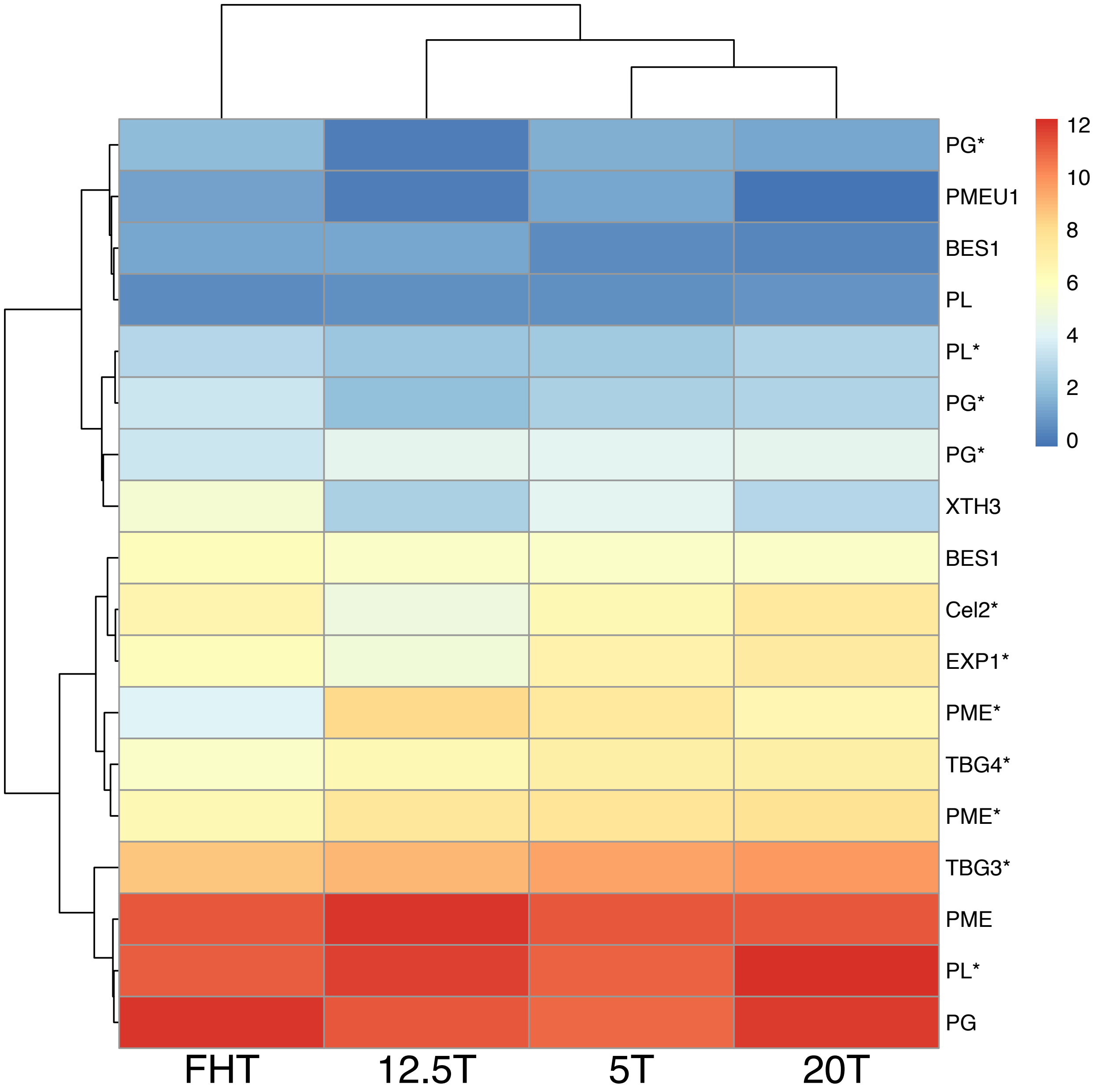

Figure S17 Transcriptomic analysis in fruit cell wall pathway. The gene expression data is Log2 CPM (counts per million) in the RNASeq data from this work, and the asterisks were added to differentially expressed genes ($p < 0.05$).

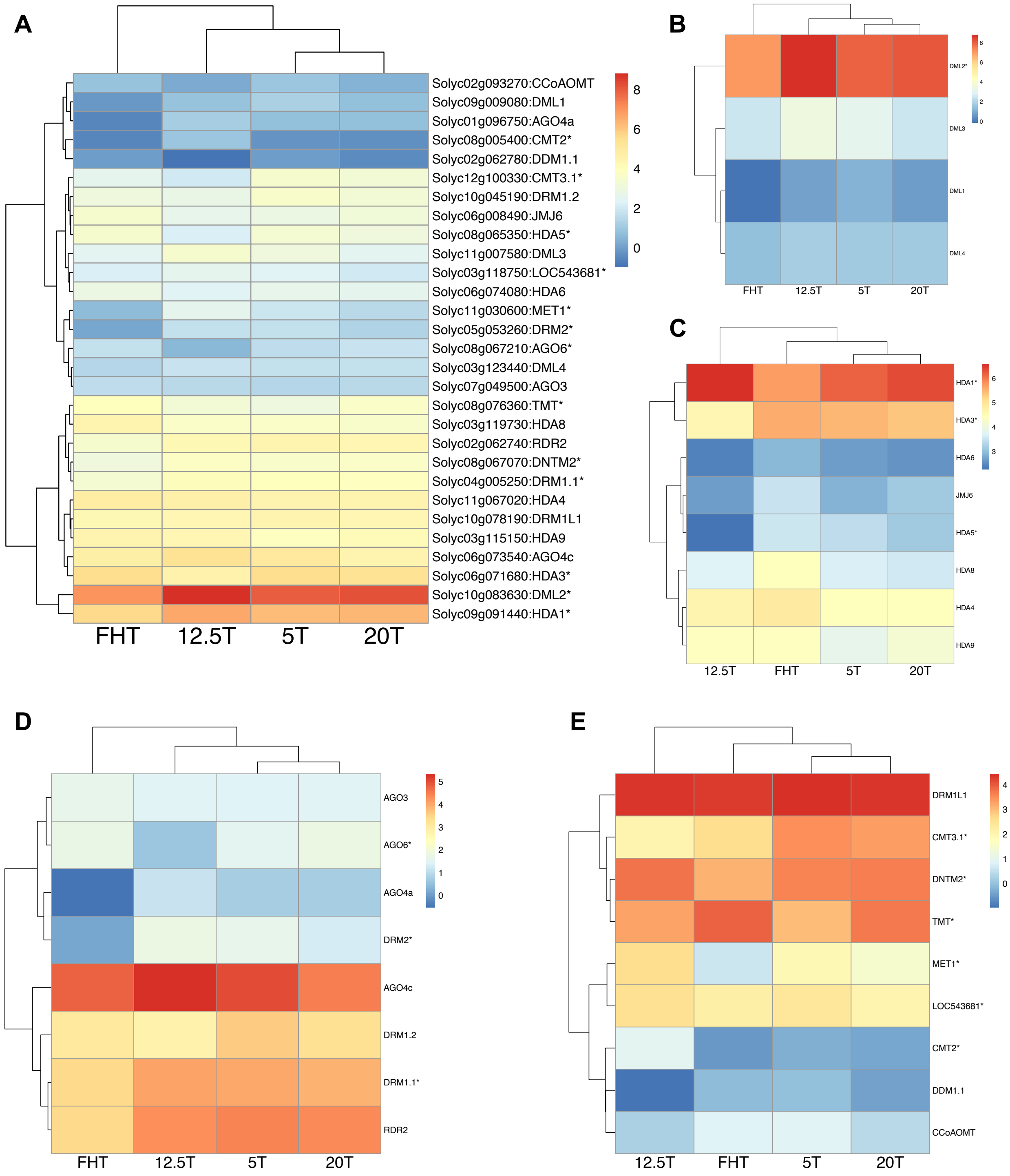

Figure S18 Transcriptomic analysis in DNA methylation and histone related pathways. **(A)** Overall DNA methylation and histone related expressed in the tomato fruit, which includes **(B)** Demethylation related, **(C)** Histone related, **(D)** RNA-directed DNA methylation (RdDM) pathway and **(E)** Methylation maintenance and methyltransferase. The gene expression data is

Log$_2$ CPM (counts per million) in the RNASeq data from this work, and the asterisks were added to differentially expressed genes ($p < 0.05$).

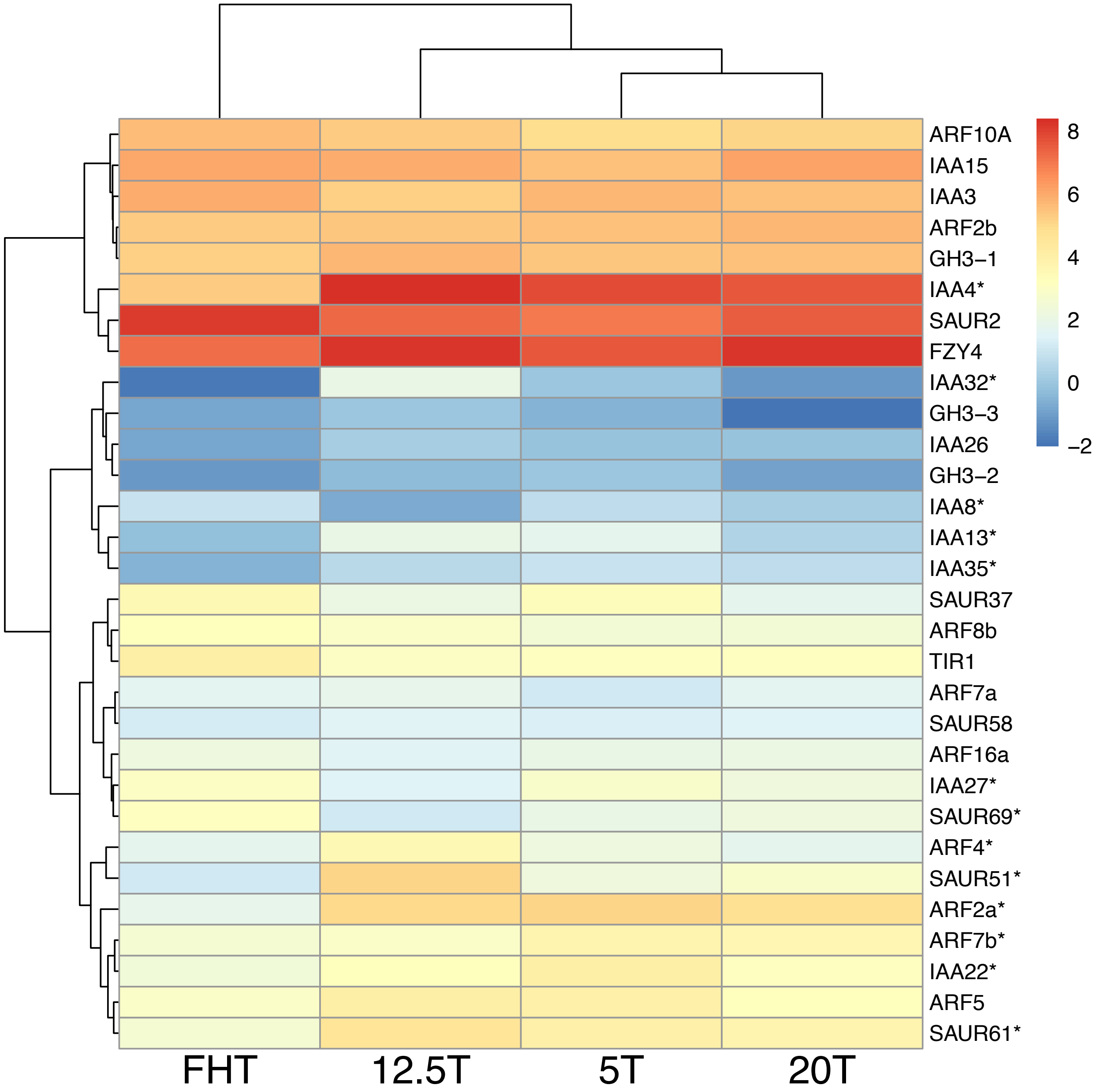

Figure S19 Transcriptomic analysis in Auxin/IAA related genes. The asterisks were added to differentially expressed genes ($p < 0.05$).

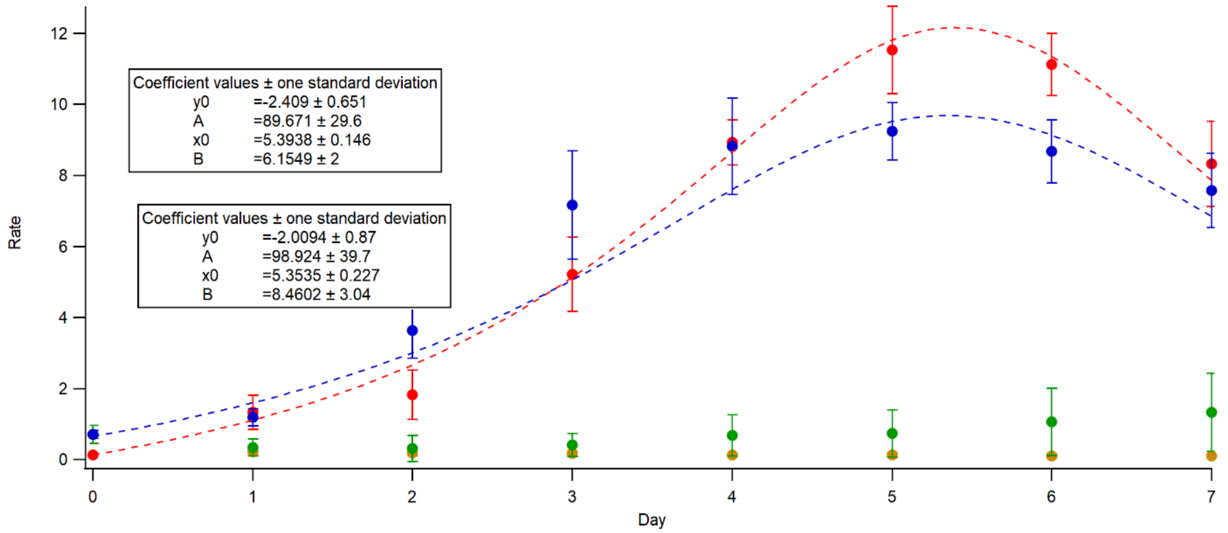

Figure S20 Ethylene production fitting curves. Ethylene of the fruit harvested at the MG and stored at 20°C (blue line) and 5°C chilled and rewarmed to 20°C (red line). The 12.5°C (green dots) and 5°C (yellow dots) were also shown, but due to the low production they are not fitted into a curve.

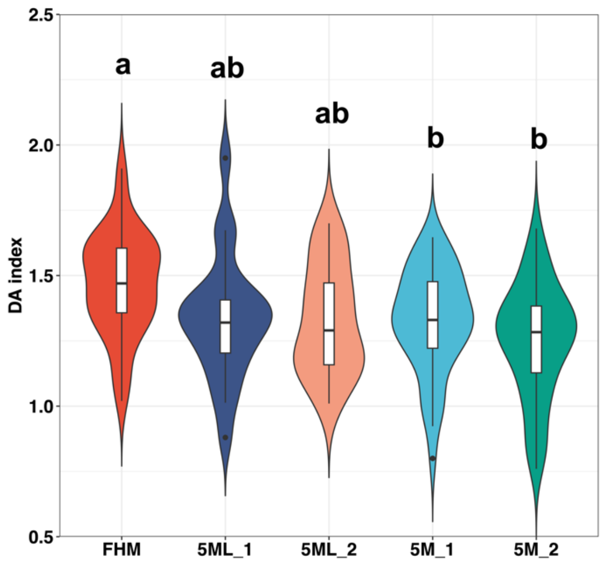

Figure S21 Postharvest green fruit DA index. ‘5M’- mature green fruit were chilled for two weeks in the dark condition , and the ‘5ML’ were supplemented with the normal white light. The

DA measurement of the ‘5M’ and ‘5ML’ were repeated shown as ‘5M_1’, ‘5M_2’, ‘5ML_1’ and ‘5ML_2’. The letters above indicate the significant difference ($p < 0.05$).

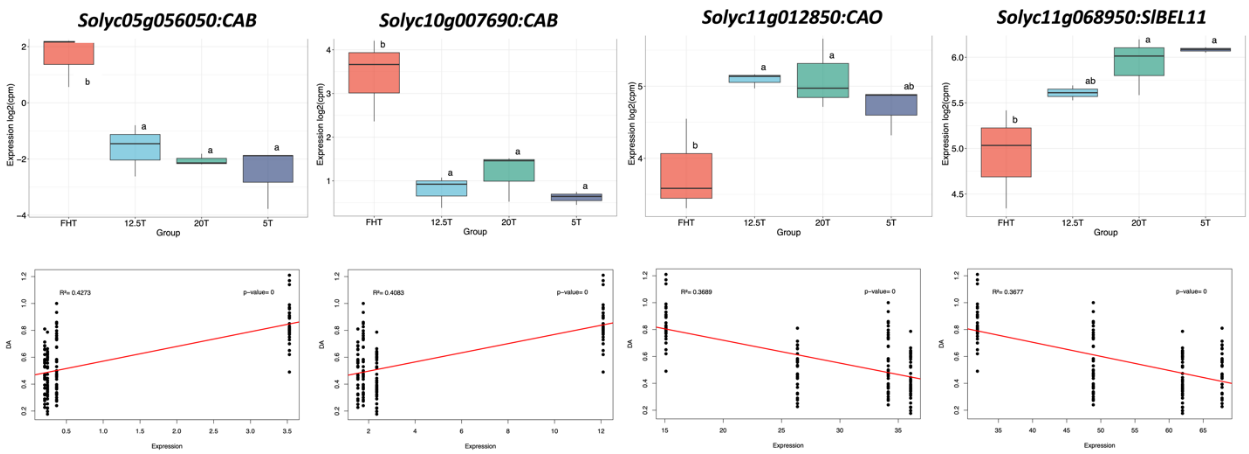

Figure S22 Photosynthetic genes with correlation between gene expression and DA index. The four columns are for the individual gene’s expression box plot and correlations for DA index and gene expression.

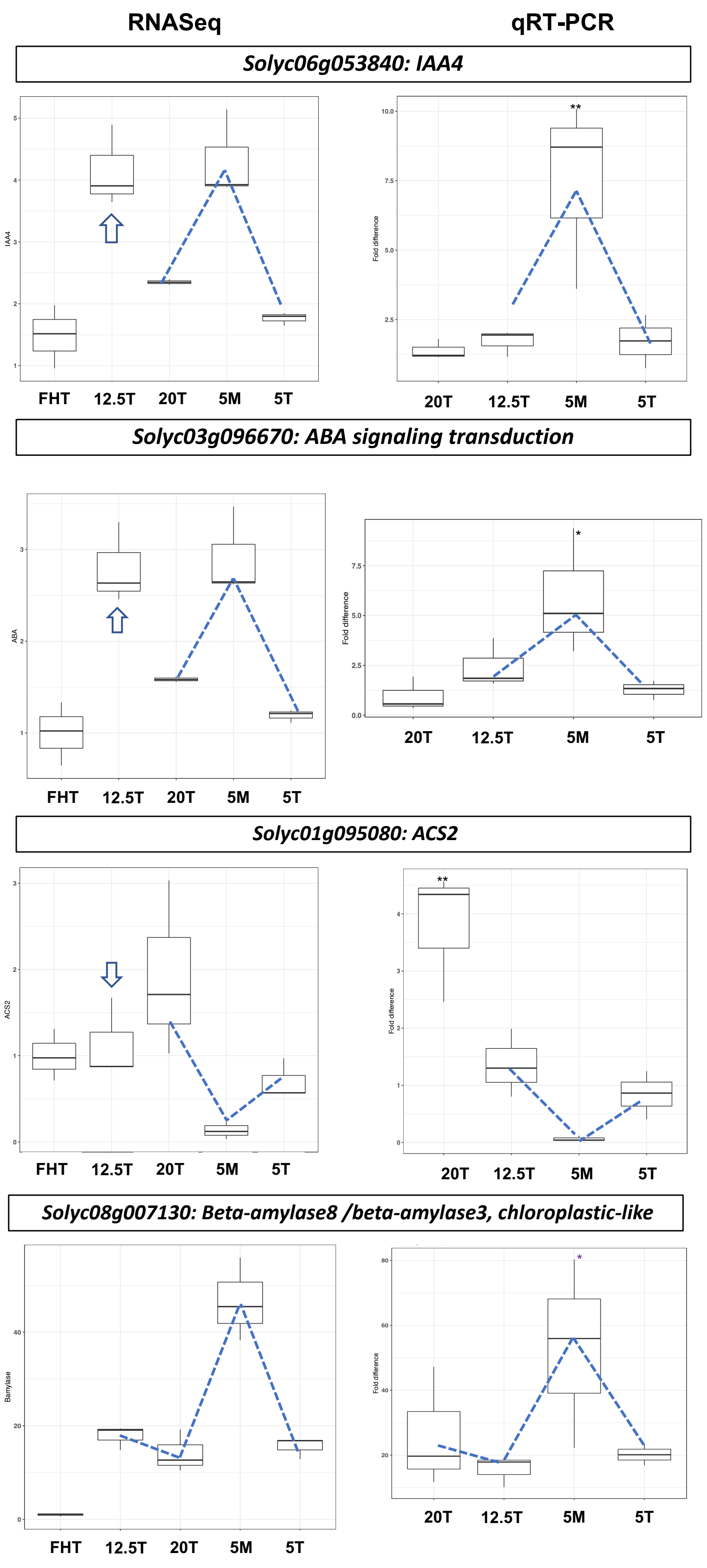
RNASeq
qRT-PCR
Solyc06g053840: IAA4
IAA4
Fold difference
FHT 12.5T 20T 5M 5T
20T 12.5T 5M 5T
Solyc03g096670: ABA signaling transduction
ABA
Fold difference
FHT 12.5T 20T 5M 5T
20T 12.5T 5M 5T
Solyc01g095080: ACS2
ACS2
Fold difference
FHT 12.5T 20T 5M 5T
20T 12.5T 5M 5T
Solyc08g007130: Beta-amylase8 /beta-amylase3, chloroplastic-like
Bamylase
Fold difference
FHT 12.5T 20T 5M 5T
20T 12.5T 5M 5T

Figure S23 RT-qPCR validation of the selected DEGs from RNASeq. Single asterisk (*) and double asterisks (**) refer to significant differences between ‘FHT’ and the postharvest treatment with *p-value* < 0.05 and *p-value* < 0.01, FHT were set as 1. The blue dashed lines indicate the trend of gene expression across samples. Compared to the ‘FHT’, the ‘20T’, ‘5M’ and ‘5T’ groups are highly consistent between the two analysis methods, while the ‘12.5T’ is differentially expressed for some genes shown as the blue arrows.
